# Natural asphalt oxide-grafted carboxylic acid: a sustainable heterogeneous catalyst for synthesis of pyrano[2,3-*c*]pyrazoles and 2-amino-3-cyanopyridines in water†

**DOI:** 10.1039/d5ra03786g

**Published:** 2025-07-16

**Authors:** Shabnam Rashidi, Mohammad Soleiman-Beigi

**Affiliations:** a Department of Chemistry, Basic of Sciences Faculty, Ilam University 69315-516 Ilam Iran m.soleimanbeigi@ilam.ac.ir SoleimanBeigi@yahoo.com

## Abstract

In this study, we successfully utilized natural asphalt as a natural carbon substrate for the synthesis of a novel heterogeneous Brønsted acid nanocatalyst, Re-NA–CH_2_CO_2_H. The –COOH functional groups present on the surface of Reduced Natural Asphalt Oxide (Re-NA-oxide) serve as catalytic sites for Brønsted acid. This arrangement, in addition to increasing acidity, also expands the surface area accessible for catalytic activity, positioning Re-NA-oxide as a viable option for a range of acid-catalyzed reactions. The synthesized catalyst was characterized using various methods, including FT-IR, TGA, SEM, EDX and TEM. This catalyst was employed in the synthesis of pyrano[2,3-*c*]pyrazole and 2-amino-3-cyanopyridine derivatives through four-component reactions involving ethyl acetoacetate, hydrazine hydrate, malononitrile, and various aldehydes, as well as ammonium acetate, malononitrile, aldehydes, and ketones, respectively. The final step of the reaction mechanism involved vinylogous anomeric-based oxidation. The high acidity of the Re-NA–CH_2_CO_2_H catalyst enhanced nucleophilic attacks on electrophiles, contributing to the efficiency of the reactions. It is noteworthy that this study uses a naturally derived catalytic support, emphasizing its sustainability. This research potentially enables the coupling of nucleophiles to natural asphalt for the development of new functional materials from this renewable resource. The reaction conversion rate is significantly influenced by the electron-donating and electron-accepting groups in the reactions of pyrano[2,3-*c*]pyrazole (90–97% yield in 20–50 min) and 2-amino-3-cyanopyridine (90–97% yield in 30–50 min). Furthermore, due to the use of water as the solvent, it is easy to separate and reuse, operational simplicity, and environmentally friendly. The catalyst exhibits exceptional recyclability and retains its activity for at least five cycles, outperforming currently available catalysts in terms of yield, reaction conditions, and overall efficiency.

## Abstract 2

In this study, we successfully utilized natural asphalt as a natural carbon substrate for the synthesis of a novel heterogeneous Brønsted acid nanocatalyst, Re-NA–CH_2_CO_2_H.

## Graphical abstract

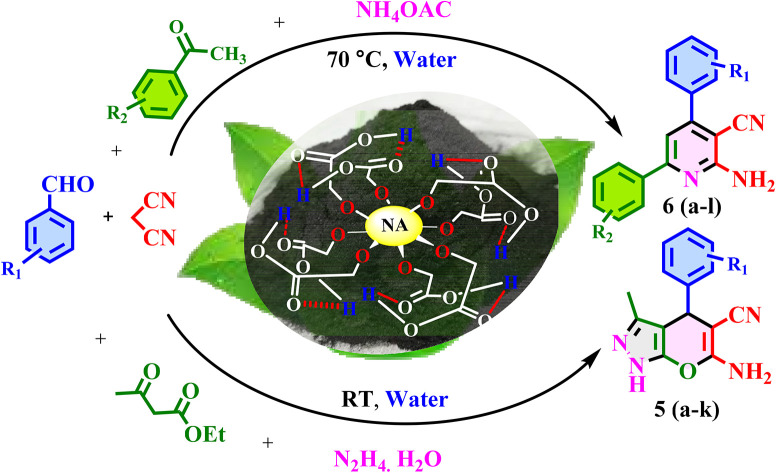

## 1. Introduction

In recent years, green chemistry has emerged as a pivotal approach for designing sustainable chemical processes that minimize environmental impact while enhancing efficiency and safety. Among the key principles of green chemistry is the development of heterogeneous catalysts capable of performing reactions under mild conditions, such as in water at room temperature and within a single reaction vessel.^1,2^ These catalysts offer significant advantages, including reduced waste generation, minimized energy consumption, and improved sustainability.^3^ Heterogeneous acid catalysts, in particular, have garnered attention due to their high reactivity, selectivity, and environmental friendliness, making them indispensable tools for advancing sustainable chemical technologies. This aligns with the principles of green chemistry by promoting the use of safer solvents, minimizing by-products, and enabling easy separation and reuse of catalysts.^4–6^

The demand for stable, cost-effective, and eco-friendly catalysts has led researchers to explore alternatives to traditional mineral acids, which are often corrosive and challenging to regenerate. Heterogeneous Brønsted acid catalysts, characterized by their low corrosiveness, higher selectivity, and ease of separation from reaction systems, represent an attractive solution.^7^ Among various types of solid acids, heteropoly acids, metal oxides, sulfonated metal oxides, phosphates, and highly acidic resins exhibit promising catalytic activity and reusability in acid-catalyzed reactions. However, their limited specific surface area restricts substrate accessibility to active sites and increases diffusion barriers.^8^

Carbon-based materials act as catalysts or supports in chemical reactions like oxidation, hydrogenation, reduction and condensation, thanks to their excellent properties such as large surface areas, high porosity, excellent electron conductivity, and relative chemical inertness. They can be modified with metallic nanoparticles to improve catalytic abilities. These materials are promising for green, solvent-free catalysis, aiming for efficient synthesis and energy use. Future advancements depend on developing eco-friendly multifunctional catalysts from nanostructured carbon.^9^

Research is continually advancing the optimization of the properties of carbon-based materials for various catalytic uses. Several Brønsted acid catalysts exist, including *p*-toluene sulfonic acid (*p*-TsOH),^10^ silica-sulfuric acid (SSA),^11^ PPF–SO_3_H,^12^ sulfonated MCM-41 (MCM–SO_3_H),^13^ a magnetic solid acid catalyst derived from chitosan (CS–Fe_3_O_4_@SO_3_H),^14^ organosilane sulfonated graphene oxide (SSi–GO)^15^ and cellulose sulfuric acid (CSA),^16^ which offer benefits such as reusability, recyclability, high yield, quick reaction times, and straightforward separation. Nevertheless, they face challenges such as costly starting materials and reagents, intricate synthesis processes, low catalytic activity, and the use of toxic solvents for product separation.^13–15^

Traditional liquid–acid catalysts, such as sulfuric acid (H_2_SO_4_), hydrochloric acid (HCl), hydrobromic acid (HBr), and trifluoroacetic acid (CF_3_COOH), often deliver high effectiveness in a range of chemical reactions, especially in various processes. However, they bring about environmental and economic issues related to the generation of waste and separation procedures. The challenges associated with separation and recovery often result in substantial quantities of non-recyclable acid waste, which not only increases disposal expenses but also poses environmental risks. To address these problems, various approaches can be utilized, such as employing solid acid catalysts, exploring alternative reaction conditions, utilizing immobilization techniques, and implementing continuous flow systems. Solid acid catalysts are becoming increasingly favored due to their non-toxic characteristics, ability to be reused, cost-effectiveness, capability of operating under gentler conditions, environmental benefits, ease of handling, stability, and versatility across a variety of chemical reactions. They enhance reactions by creating a pathway that requires lower activation energy, thus accelerating reaction rates.^17^

Acids play a key role in many chemical processes in biology and industry. Recently, chiral Brønsted acids have quickly advanced for creating C–C and C–X bonds. Weakly acidic compounds like (thio)ureas, squaramides, and others that activate substrates through hydrogen bonding have become popular organocatalysts. Some of these have been used in anion binding catalysis and other new applications. Since phosphoric acids were introduced as asymmetric catalysts in 2004, Brønsted acid catalysis has gained attention for asymmetric synthesis.^18,19^ Carboxylic acids are common in organic chemistry and found in natural compounds. However, their use for substrate activation is less developed due to their weaker acidity, which limits the types of substrates that can be activated. However, selecting an acid catalyst with suitable acidity can be crucial for the effective activation of specific types of substrates.^19,20^

Heterogeneous Brønsted carboxylic acid catalysts, including simple, aromatic, long-chain, mixed carboxylic acids, and those with mineral structures, play a crucial role in diverse chemical transformations, ranging from the synthesis of pharmaceuticals and biological compounds to industrial chemicals. Their significance in green chemistry lies in their ability to optimize chemical processes, reduce energy consumption, minimize waste production, and enhance product quality.^7,19,21^

**Fig. 1:**
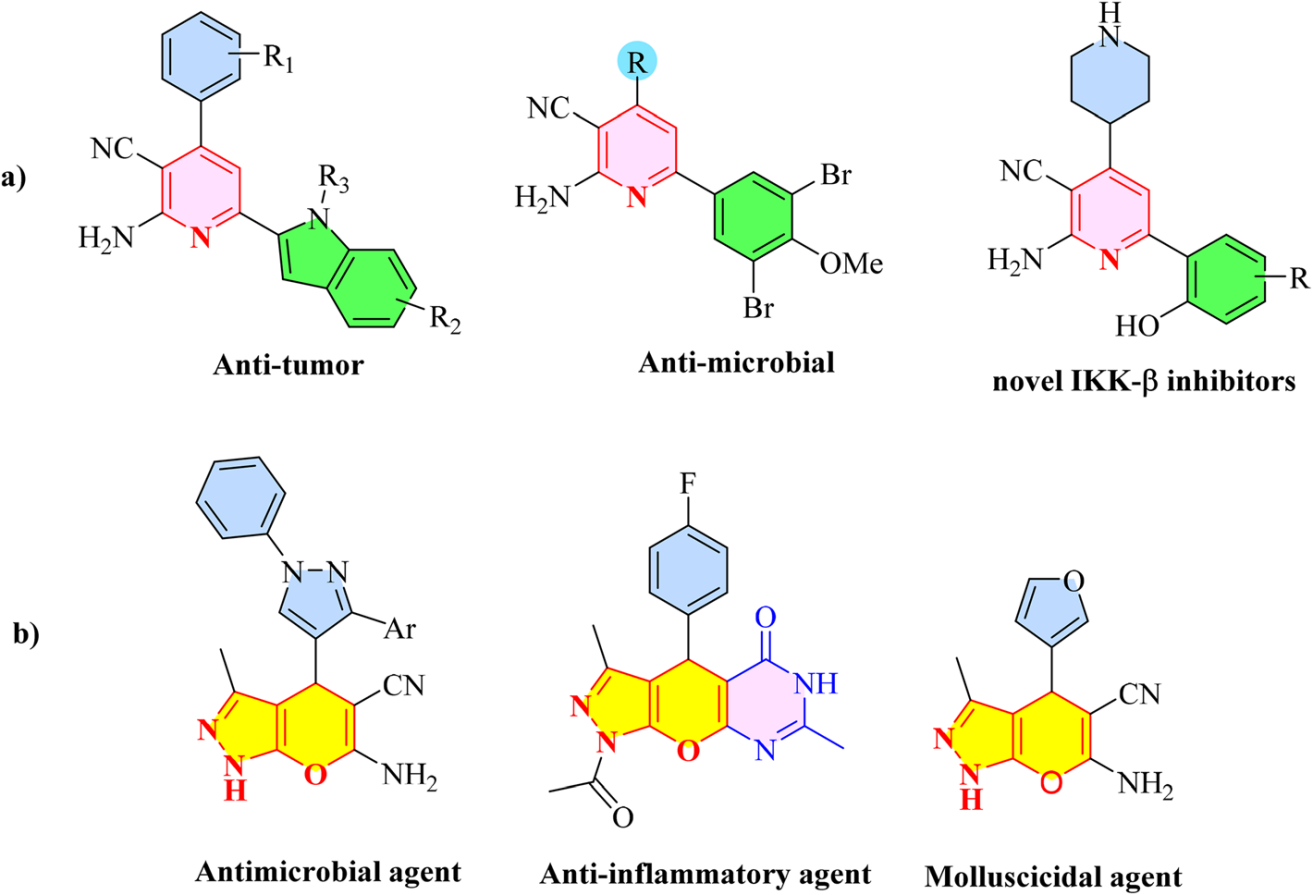
Biological properties (a) 2-amino-3-cyanopyridine (b) pyrano[2,3-*c*]pyrazole.

Multicomponent reactions (MCRs) have become increasingly important in modern synthetic chemistry due to their atom-efficient properties and ability to effectively construct complex molecular structures. MCRs offer an effective and environmentally friendly method for synthesis. Using sustainable solvents with MCRs helps reduce waste and improve safety. MCRs combine materials in one step to create final products, known for their efficiency, convergence, and high atom economy. By combining three or more reactants in a single step, MCRs provide a powerful tool for combinatorial synthesis, significantly increasing molecular diversity and complexity. The most important subclasses of heterocyclic chemistry are oxygen and nitrogen containing rings that are found in the skeletal structures of a variety of biologically active and pharmaceutical compounds.^8,22–24^ Among the oxygen and nitrogen containing heterocycles, pyrano[2,3-*c*]pyrazoles and 2-amino-3-cyanopyridines have anticancer, anticoagulant, anticonvulsant, antimicrobial, anti-HIV, antimalarial, antitumor, antibacterial, antifungal, and antitumor properties. Also 2-amino-3-cyanopyridines exhibit potent activities as IKK-β inhibitors and adenosine A_2A_ receptor antagonists (Fig. 1).^25^

Natural asphalt, also known as uintaite or asphaltum, is a resinous hydrocarbon created over millions of years through geological processes. It mainly contains hydrocarbons, asphaltenes, resins, and minerals, and is highly soluble in organic solvents like trichloroethylene, carbon disulfide, and toluene. Natural asphalt consists of 70–80% carbon and about 15% hydrogen, with small amounts of oxygen, sulfur, and nitrogen. Key sources are in the U. S., Canada, and Iran. Its non-toxic nature allows for uses in producing coke, asphalt, dyes, drilling mud, and foundry industries, with research exploring its properties in organic reactions. Its benefits include availability, affordability, stability, and high carbon content, making it effective as a carbon support for catalysts. Often used in powder form due to its brittleness, natural asphalt is a candidate for creating sustainable materials, including heterogeneous catalysts.^26–28^

Continuing previous research, the aim of this study is to synthesize a new heterogeneous Brønsted acid catalyst derived from natural asphalt oxide and investigate its catalytic activity in the synthesis of multicomponent reactions pyrano[2,3-*c*]pyrazoles and 2-amino-3-cyanopyridines (Schemes 2 and 3) in line with the principles of green chemistry in aqueous solvent and mild conditions.

In this study, we present a significant innovation in the synthesis of a novel heterogeneous Brønsted acid nanocatalyst, Re-NA–CH_2_CO_2_H, using natural asphalt as a stable carbon support. The presence of –COOH functional groups on the surface of reduced natural asphalt oxide (Re-NA-oxide) increases the acidity and increases the surface area available for catalytic activities, making it an effective option for a variety of acid-catalyzed reactions. The high acidity of the catalyst facilitates nucleophilic attacks on electrophiles, thereby improving the overall efficiency of the reactions. Key advantages of this catalyst include high catalytic activity, operational simplicity, and environmental friendliness due to the use of water as a solvent.

## 2. Experimental

### 2.1. Materials and reagents

#### 2.1.1. Reagents and synthesis of Brønsted acid catalyst Re-NA–CH_2_CO_2_H

The chemicals and solvents used in this study were supplied by pharmaceutical companies Merck and Sigma-Aldrich. The chemicals including sulfuric acid (H_2_SO_4_) (98%), potassium permanganate (KMNO_4_) (99%), sodium nitrate (NaNO_3_) (99.5%), hydrochloric acid (HCl) (37%), hydrogen peroxide (H_2_O_2_) (30%), hydrazine hydrate (N_2_H_4_. H_2_O) (100%), sodium hydride (NaH), ethanol (96%), tetrahydrofuran (THF) (99.8%), chloroacetic acid (ClCH_2_CO_2_H) (99%) were purchased from Merck chemical company and natural asphalt powder (mesh 150–200) was provided by mines of Ilam.

**Scheme 1:**
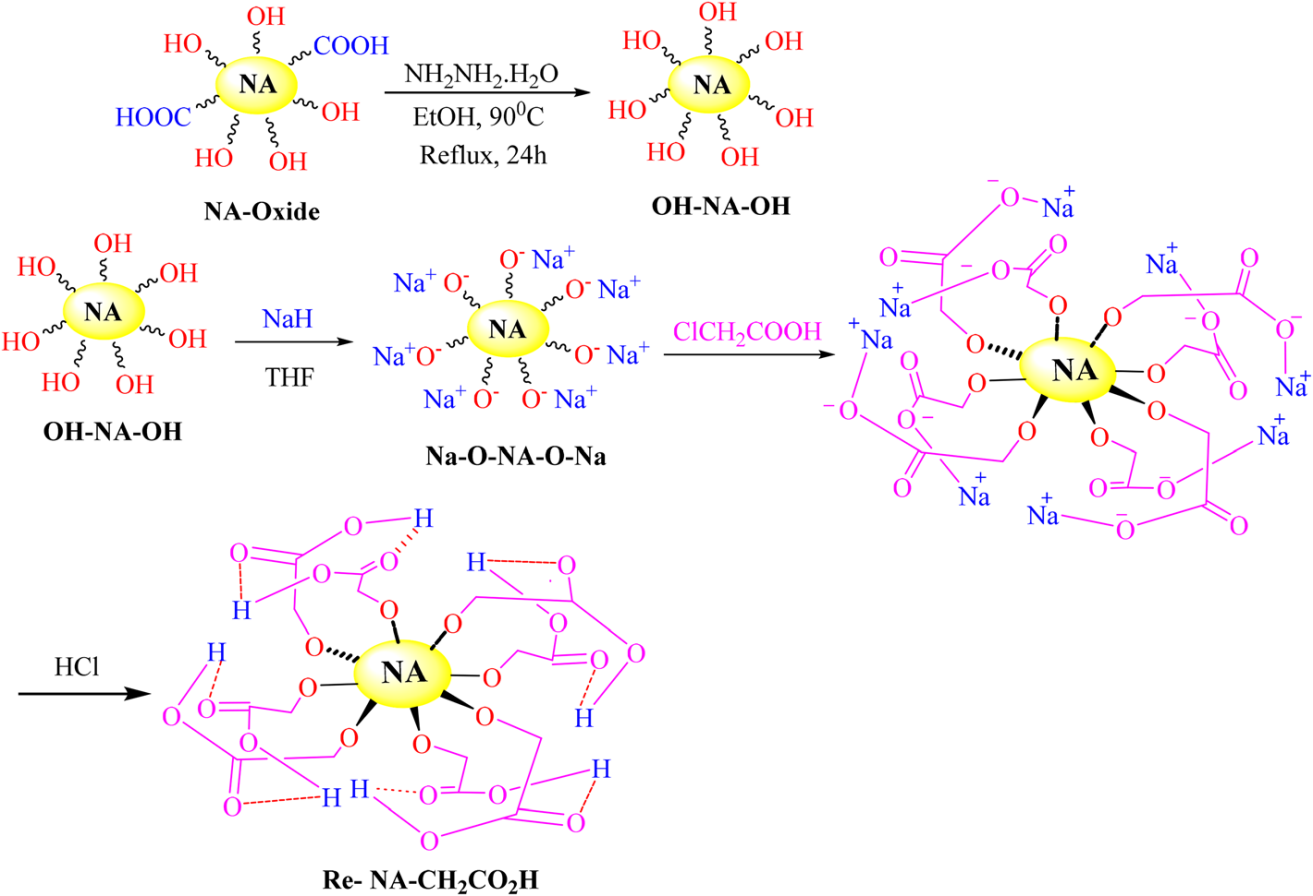
Synthesis of Brønsted acid catalyst Re-NA–CH_2_CO_2_H.

Oxidized natural asphalt (NA-oxide) was synthesised utilizing the Hummers' method, as outlined in earlier research^27^ (Scheme 1). The resulting asphalt oxide underwent Soxhlet extraction with water for a duration of 24 hours. Subsequent to this, the NA-oxide was reduced using hydrazine hydrate. This procedure involved dispersing 0.3 g of NA-oxide in 50 mL of ethanol and placing the mixture in an ultrasonic bath for 30 minutes to ensure thorough exfoliation and uniform distribution. Following this, 2.5 mL of hydrazine hydrate was introduced into the solution, and the mixture was stirred under reflux conditions for 24 hours to promote the reduction reaction. Upon completion of the reaction, the black precipitate of reduced asphalt oxide (Re-NA-oxide) was collected through vacuum filtration on filter paper. The precipitate was then washed multiple times with distilled water and ethanol to eliminate any unreacted substances or by-products. Ultimately, the product was dried in an oven at 50 °C. Next, 0.3 g of Re-NA-oxide was combined with 30 mL of tetrahydrofuran (THF) as a solvent and 0.15 g of sodium hydride (NaH) in a 100 mL flask. The mixture was stirred for 30 minutes, after which 0.3 g of chloroacetic acid was added. The reaction continued for 24 hours under reflux with ongoing stirring. Subsequently, 2 mL of 37% HCl was incorporated, and the mixture was stirred for an additional 2 hours. The resulting precipitate was filtered, thoroughly washed with water and ethanol, and then dried in an oven at 50 °C. The FT-IR spectrum of the final precipitate was recorded for characterization purposes.

### 2.2. Instrumental

Fourier-transform infrared spectroscopy (FT-IR) was recorded as KBr pellets using FT-IR, VERTEX 70, Bruker, Germany spectroscopy. The morphology and size of Re-NA–CH_2_CO_2_H catalyst particles were examined using scanning electron microscope (SEM) Mira 3-XMU and elemental analysis of Re-NA–CH_2_CO_2_H was performed by energy dispersive X-ray (EDX), TESCAN MIRA, Czech Republic. Thermal analysis (TGA) and differential scanning calorimetry (DSC) were performed using a PerkinElmer-STA6000 apparatus, USA. The analysis of transmission electron microscopy (TEM) was performed with Philips-EM 208S TEM. Proton nuclear magnetic resonance (^1^H NMR) spectroscopy data of all the synthesized compounds were recorded using a Bruker DRX-250 AVANCE instrument in DMSO-d_6_ as the solvent and tetramethylsilane (TMS) as an internal reference. Melting points were determined in capillary tubes using Barnstead Electrothermal 9100 and the reaction progress and the purity of the products were monitored by thin layer chromatography (TLC) on aluminum coated.

### 2.3. Catalytic properties of Brønsted acid catalyst Re-NA–CH_2_CO_2_H

#### 2.3.1. Synthesis of pyrano[2,3-*c*]pyrazole derivatives

**Scheme 2:**
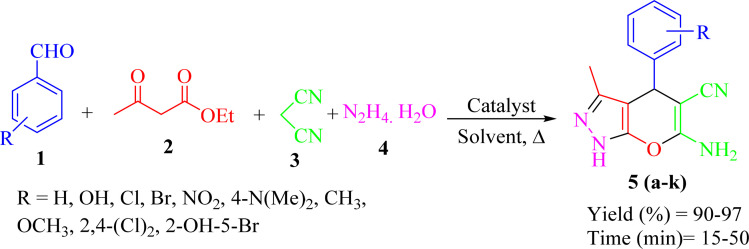
General procedure for the synthesis of pyrano[2,3-*c*]pyrazole.

In a test tube, aldehyde 1 (1 mmol) ethyl acetoacetate 2 (1 mmol), malononitrile 3 (1 mmol), hydrazine hydrate 4 (1 mmol), were mixed in the presence of 10 mg of Re-NA–CH_2_CO_2_H. The mixture was stirred in water at 25 °C for a specified period of time. The progress of the reaction was monitored by thin-layer chromatography (TLC) with a solvent system of *n*-hexane and ethyl acetate (8 : 2 ratio). Upon completion of the reaction, the catalyst was separated by filtration using hot ethanol. The solvent was then evaporated, and the resulting solid product was recrystallized from hot ethanol. The final products were characterized and confirmed by ^1^H NMR and ^13^C NMR spectroscopy (Scheme 2).

#### 2.3.2. Synthesis of the four-component one-pot 2-amino-3-cyanopyridine derivatives

**Scheme 3:**
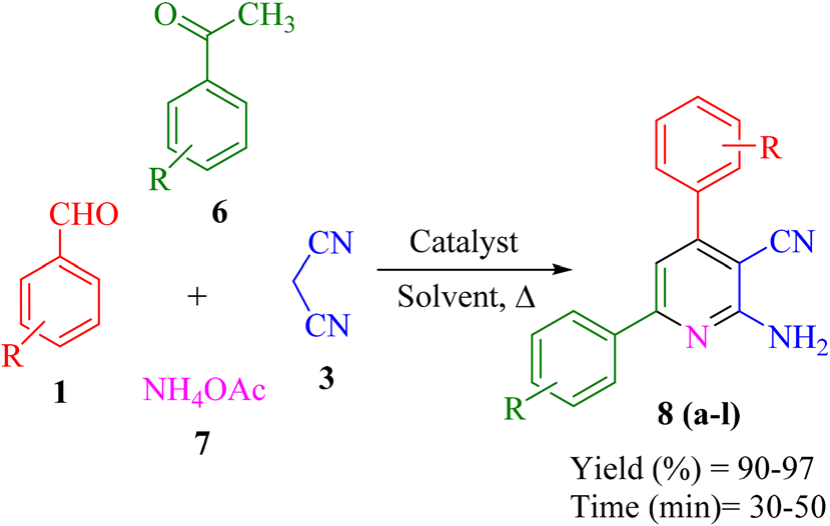
General procedure for the synthesis of 2-amino-3-cyanopyridine.

In a test tube, a mixture of aromatic aldehydes 1 (1 mmol), acetophenone 6 (1 mmol), malononitrile 3 (1 mmol), ammonium acetate 7 (1 mmol) as the nitrogen source, and Re-NA–CH_2_CO_2_H (15 mg) was added. The mixture was stirred in water at 70 °C using an oil bath, and the reaction progress was monitored by thin-layer chromatography (TLC) with a solvent system of *n*-hexane and ethyl acetate (7 : 3 ratio). Upon completion of the reaction, the catalyst was easily separated by filtration using hot ethanol. The solvent was then evaporated, and the resulting solid product was recrystallized from hot ethanol. The final products were characterized and confirmed by ^1^H NMR and ^13^C NMR spectroscopy (Scheme 3).

## 3. Results and discussion

The synthesis process began with the oxidation of natural asphalt using Hummers' method, followed by its reduction with hydrazine hydrate to yield reduced asphalt oxide. In the subsequent step, chloroacetic acid was immobilized onto the reduced asphalt oxide, leading to the successful synthesis of the Brønsted acid catalyst, Re-NA–CH_2_CO_2_H. The structural and morphological properties of the synthesized catalyst were thoroughly characterized using a combination of advanced analytical techniques.

Fourier-transform infrared spectroscopy (FT-IR) was employed to confirm the functional groups present in the catalyst, providing evidence for the successful immobilization of chloroacetic acid. Scanning electron microscopy (SEM) and transmission electron microscopy (TEM) were utilized to examine the morphology and particle size distribution of the catalyst, revealing a well-defined and uniform structure. Thermal stability was assessed through thermogravimetric analysis (TGA), which demonstrated the catalyst's resilience under elevated temperatures. Additionally, energy-dispersive X-ray spectroscopy (EDX) was performed to determine the elemental composition, further confirming the successful incorporation of chloroacetic acid into the catalyst framework.

These comprehensive analyses not only validated the successful synthesis of Re-NA–CH_2_CO_2_H but also provided detailed insights into its structural and thermal properties, laying a solid foundation for its application in catalytic processes.

### 3.1. Synthesis and catalyst characterization

After the successful synthesis of Re-NA–CH_2_CO_2_H, the structure characteristics of this heterogeneous Brønsted acid catalyst were thoroughly confirmed using FT-IR, SEM, TEM, EDX, TGA techniques.

#### 3.1.1. FT-IR studies

**Fig. 2:**
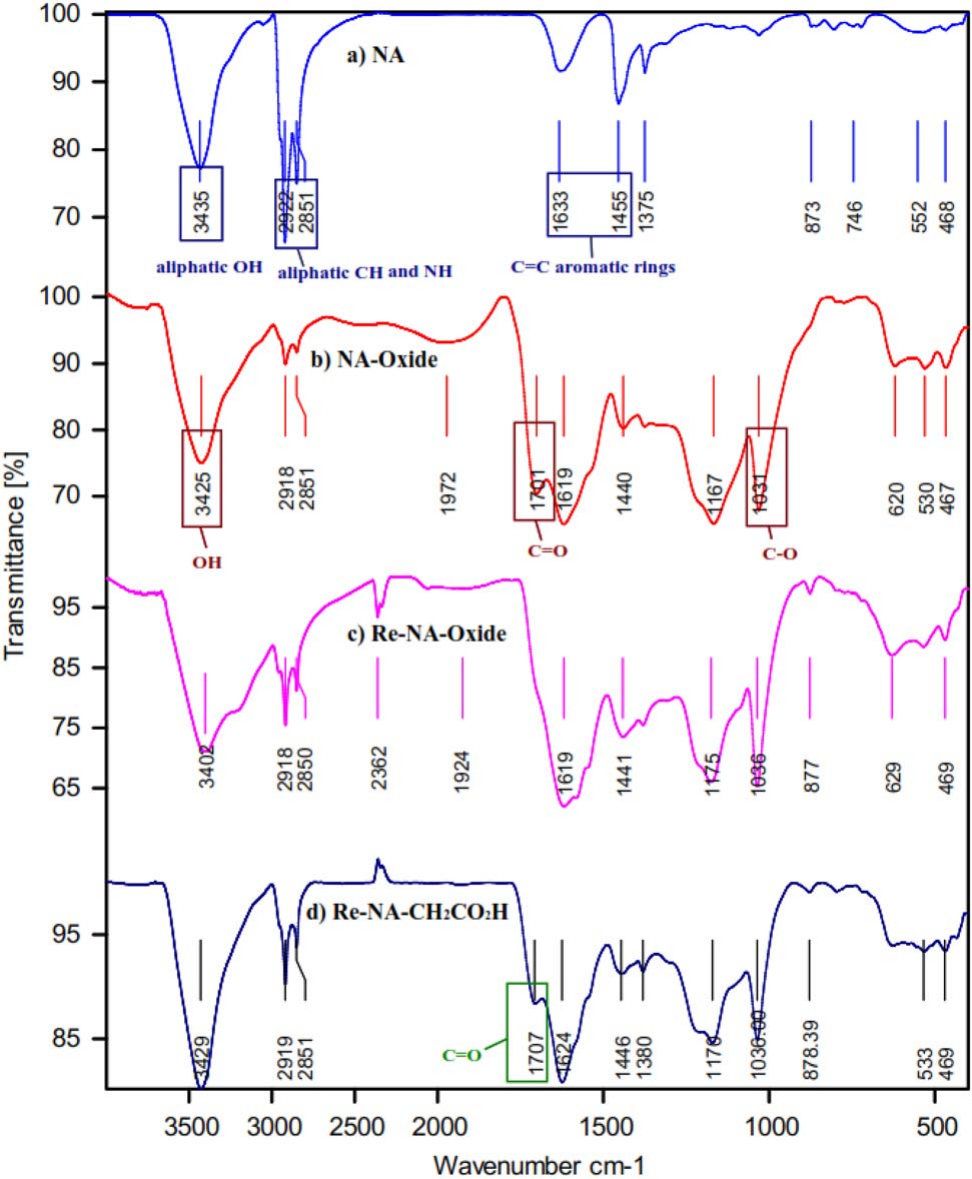
FT-IR spectra of (a) NA (b) NA-oxide (c) Re-NA-oxide (d) Re-NA–CH_2_CO_2_H.

The FT-IR spectrum of NA (a), NA-oxide (b), Re-NA-oxide (c) and Re-NA–CH_2_CO_2_H (d) are presented in Fig. 2. In spectra (a), the peaks observed in the 1445–1633 cm^−1^ are attributed to the aromatic rings in the structure of natural asphalt. Additionally, stretching vibrations related to aliphatic C–H and NH and OH groups of natural asphalt appeared in the region of 2851–2922 and 3435 cm^−1^, respectively. In spectra (b), C–O, C=O and OH stretching vibrations of carboxylic acid are shown in the region of 1031, 1701 and 3425 cm^−1^, respectively, indicates the oxidation of aromatic and aliphatic parts (asphaltene) in natural asphalt.^27^ In spectrum (c), the disappearance of the carbonyl peak at 1701 cm^−1^ confirms the successful reduction of asphalt oxide. Finally, in spectrum (d), the peak at 1707 cm^−1^ corresponds to the carbonyl group of the acidic moiety in chloroacetic acid, confirming its immobilization on the reduced asphalt oxide.

#### 3.1.2. TGA analysis

**Fig. 3:**
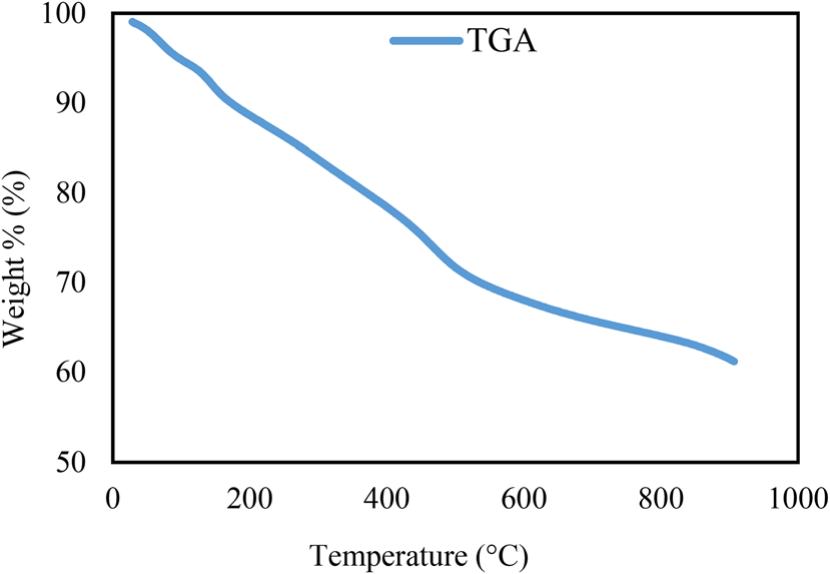
TGA analysis curves of Brønsted acid nanocatalyst Re-NA–CH_2_CO_2_H.

The TGA curve of the Re-NA–CH_2_CO_2_H catalyst, as shown in Fig. 3, demonstrates a continuous weight loss with increasing temperature, indicating its thermal decomposition behavior. The weight loss occurs in two distinct stages. The first stage, observed below 200 °C, corresponds to the removal of physically adsorbed solvents and moisture from the catalyst surface. The second stage, occurring above 200 °C, is attributed to the decomposition of organic functional groups within the catalyst structure. Notably, the catalyst retains approximately 70% of its initial mass even at a high temperature of 700 °C, highlighting its exceptional thermal stability. This property makes the Re-NA–CH_2_CO_2_H catalyst suitable for applications requiring high-temperature conditions.

#### 3.1.3. SEM analysis

**Fig. 4:**
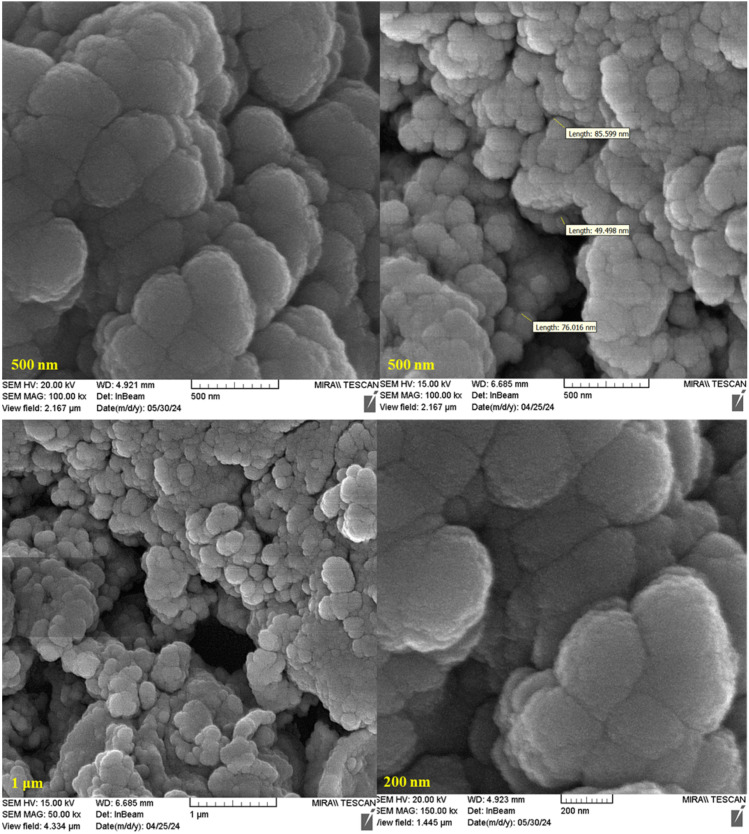
SEM images of the Brønsted acid nanocatalyst Re-NA–CH_2_CO_2_H.

The surface morphology of the synthesized catalyst Re-NA–CH_2_CO_2_H was investigated using scanning electron microscope (SEM). As shown in Fig. 4 and S1,† SEM images were captured at different magnifications to provide detailed insights into the structure of the catalyst. The obtained images reveal that the nanoparticles are spherical in shape and fall within the nano-size range, confirming the successful synthesis of nanoscale materials. This characteristic is crucial for enhancing the catalytic activity due to the increased surface area and active sites available for reactions.

#### 3.1.4. TEM analysis

**Fig. 5:**
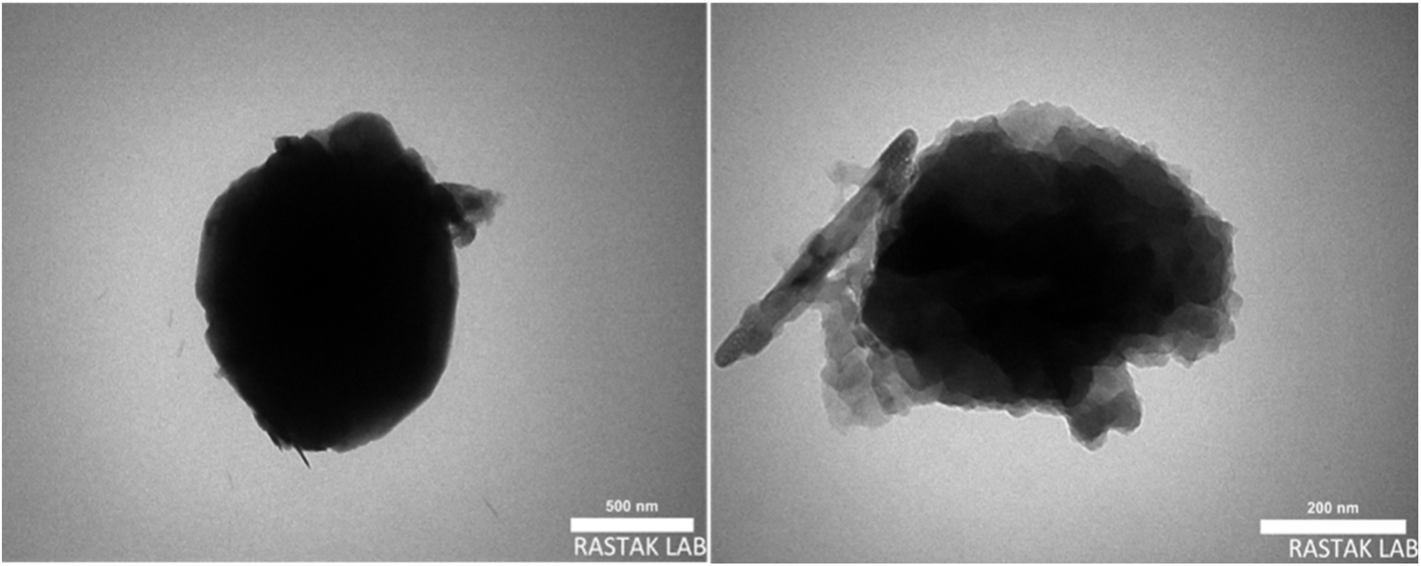
TEM images of the Brønsted acid nanocatalyst Re-NA–CH_2_CO_2_H.

The shape and size particle of the synthesized catalyst Re-NA–CH_2_CO_2_H were studied by transmission electron microscopy (TEM). As shown in Fig. 5 and S2,† the TEM images confirm the formation of particles with a spherical morphology. This characterization technique provides clear evidence of the nanoscale structure and uniformity of the synthesized material, supporting its potential application as an efficient heterogeneous catalyst.

#### 3.1.5. EDX analysis

**Fig. 6:**
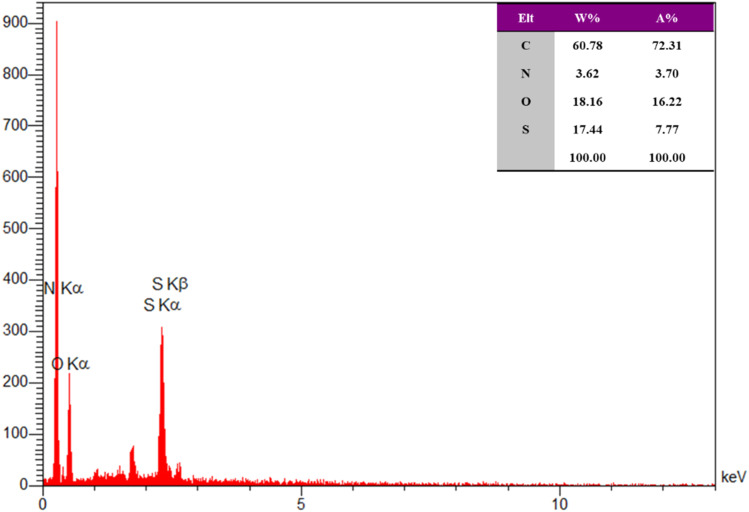
EDX analysis of the Brønsted acid nanocatalyst Re-NA–CH_2_CO_2_H.

The energy dispersive X-ray (EDX) analysis is valuable techniques for characterizing the elemental composition of materials, including heterogeneous catalysts. In this study, the EDX spectrum of the synthesized catalyst Re-NA–CH_2_CO_2_H, presented in Fig. 6, confirms the presence of key elements carbon (C), oxygen (O), nitrogen (N), and sulfur (S) within its structure. This elemental composition supports the successful synthesis of the catalyst and highlights its complex architecture.

### 3.2. Catalytic properties of Re-NA–CH_2_CO_2_H

#### 3.2.1. Synthesis of pyrano [2,3-*c*]pyrazole derivatives

To obtain the optimal conditions for the synthesis of pyrano[2,3-*c*]pyrazole derivatives, a four-component reaction between benzaldehyde (1 mmol), ethyl acetoacetate (1 mmol), hydrazine hydrate (1 mmol) and malononitrile (1 mmol) was chosen as the sample reaction and various parameters, including the amount of catalyst, reaction temperature and different solvents were studied. The results are shown in Table 1.

**Table 1:** Optimization synthesis of pyrano[2,3-*c*]pyrazole in the presence of Re-NA–CH_2_CO_2_H^a^

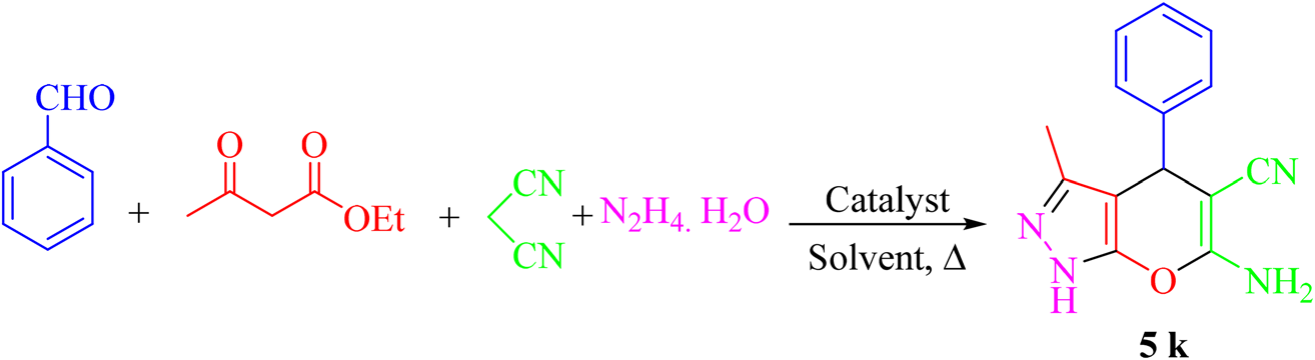				
Entry	Catalyst (mg)	Solvent	Temperature (°C)	Yield^b^ (%)
1	30	Water	90	90
2	30	Ethanol	Reflux	87
3	30	Water : ethanol (1 : 1)	90	89
4	30	Ethyl acetate	Reflux	87
5	30	Dimethylformamide	90	88
6	30	Water	70	92
7	30	Water	50	95
8	30	Water	25	95
9	15	Water	25	95
**10**	**10**	**Water**	**25**	**97**
11	10	Water	25	N.R.^c^
12	10	Water	25	30^d^
13	5	Water	25	86
14	—	Water	25	N.R.
15	—	Water	90	N.P.^e^

a Conditions: benzaldehyde (1 mmol), malononitrile (1 mmol), ethyl acetoacetate (1 mmol), hydrazine hydrate (1 mmol), catalyst Re-NA–CH_2_CO_2_H (mg), solvent (3 mL) and time: 20 min.

b Isolated yield.

c Catalyst NA, 4 h.

d Catalyst NA-oxide.

e N.P.: not product.

The best performance of the product 5k is obtained by the reaction in an aqueous solvent, at room temperature and in the presence of 10 mg of Re-NA–CH_2_CO_2_H (Table 1, entry 10). To verify the catalytic activity, we extended the reaction to a series of aromatic aldehydes and ethyl acetoacetate, hydrazine hydrate and malononitrile under optimal reaction conditions, and the results are reported in Table 2. Derivatives synthesized with electron-withdrawing aldehyde groups showed higher yields compared to those with electron-donating groups.

**Table 2:** Synthesis of pyrano[2,3-*c*]pyrazole in the presence of Re-NA–CH_2_CO_2_H^a^

Entry	Product	Time (min)	TOF (min^−1^)	TON	Yield^b^ (%)	Mp (°C)	Mp (°C) ref.
1	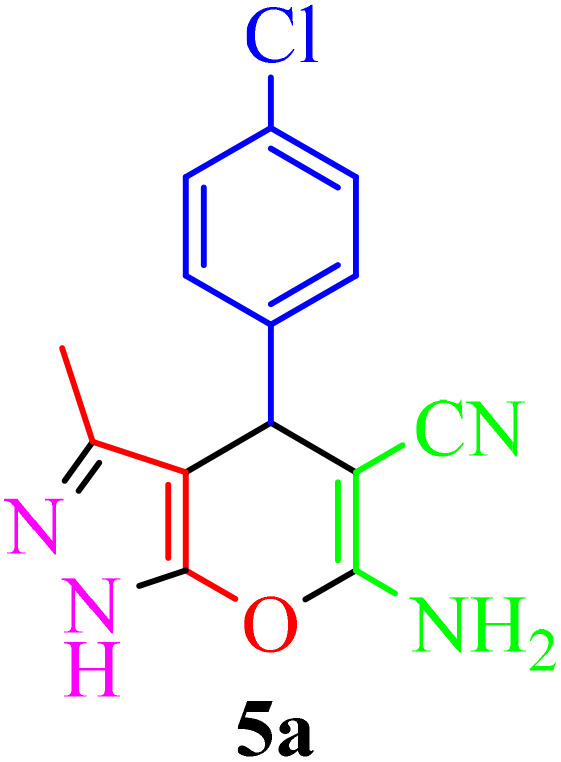	25	1.02 × 10^4^	2.54 × 10^5^	96	231–233	230–232 (ref. 29)
2	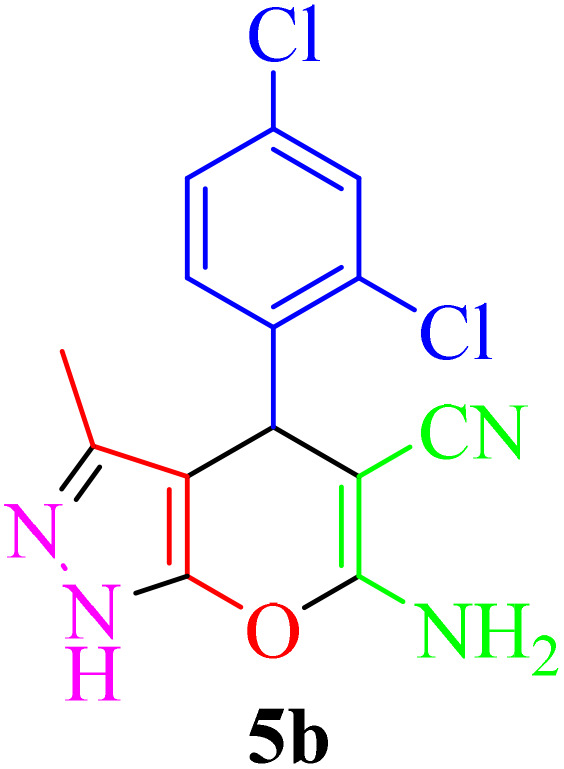	50	4.76 × 10^3^	2.38 × 10^5^	90	234–236	234–236 (ref. 29)
3	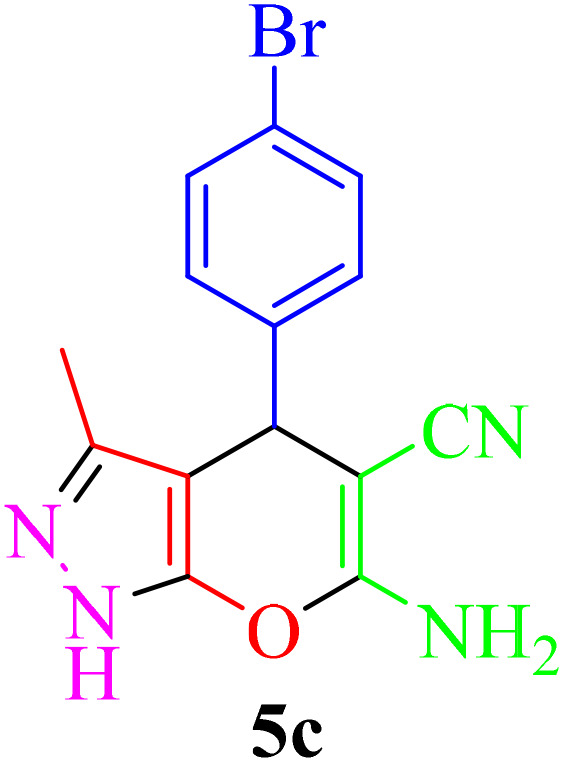	25	1.02 × 10^4^	2.54 × 10^5^	96	178–180	179–181 (ref. 30)
4	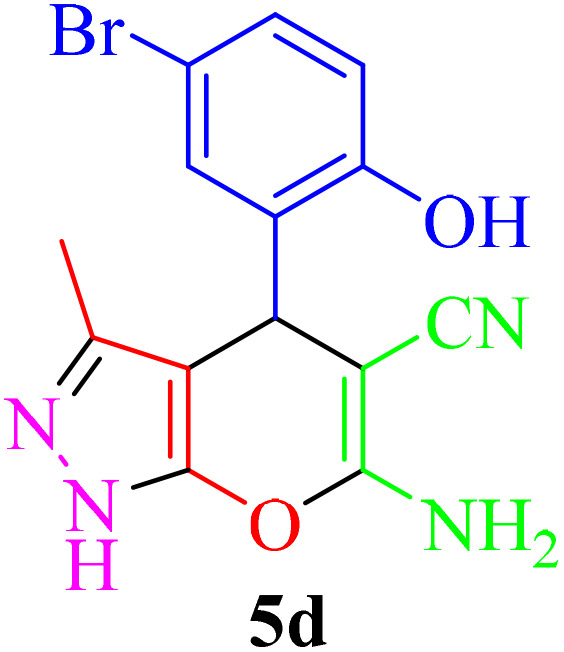	30	4.76 × 10^3^	2.38 × 10^5^	92	234–236	229–231 (ref. 31)
5	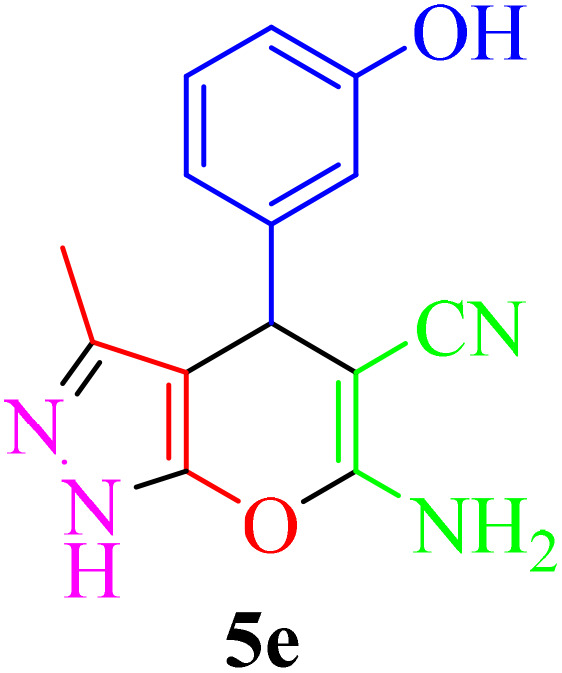	25	9.95 × 10^3^	9.95 × 10^3^	94	238–241	240–242 (ref. 32)
6	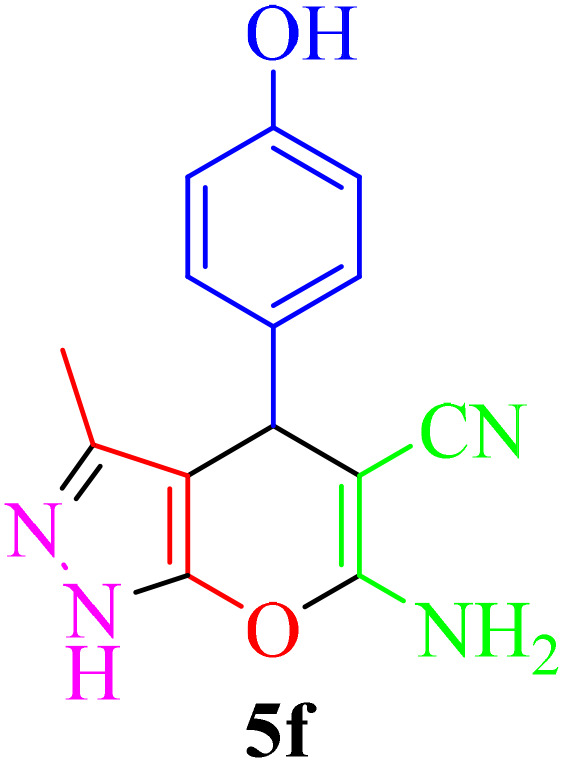	20	1.23 × 10^4^	2.46 × 10^5^	93	219–221	218–220 (ref. 29)
7	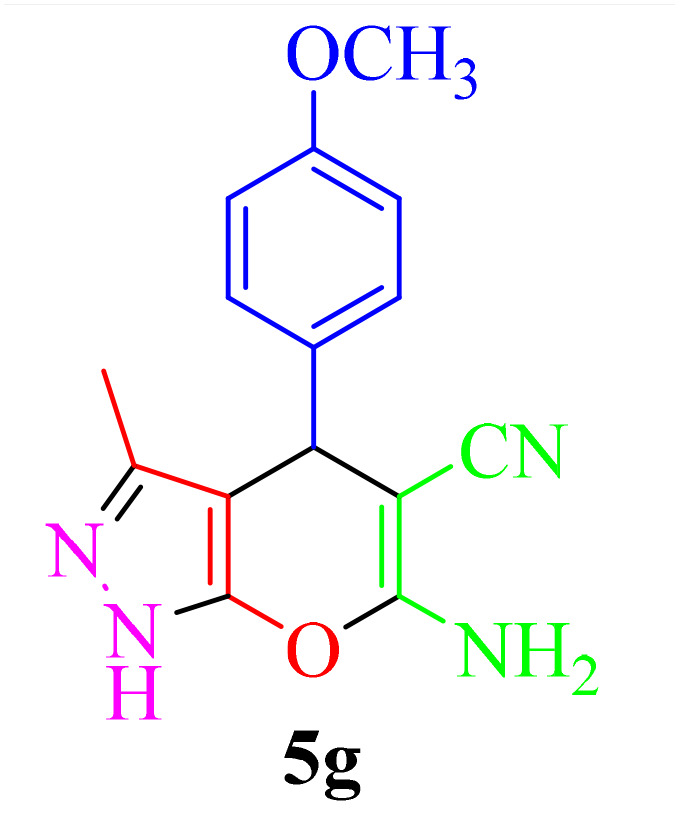	20	1.24 × 10^4^	2.49× 10^5^	94	211–213	209–211 (ref. 33)
8	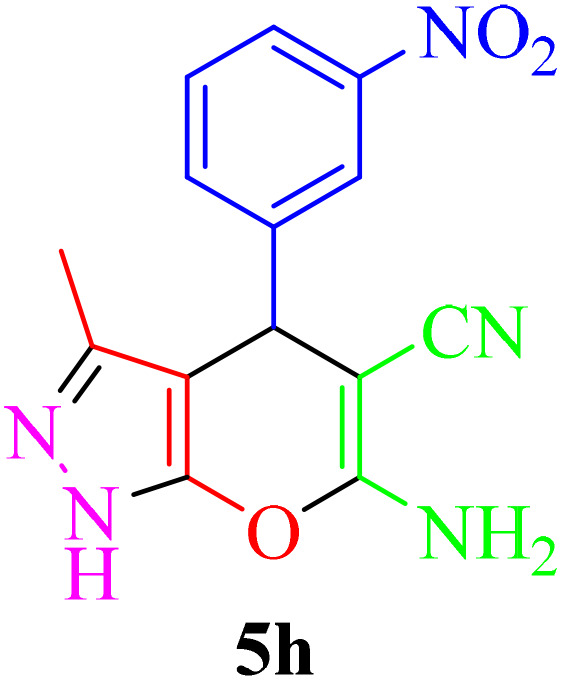	25	1.01 × 10^4^	2.51 × 10^5^	95	215–217	214–216 (ref. 34)
9	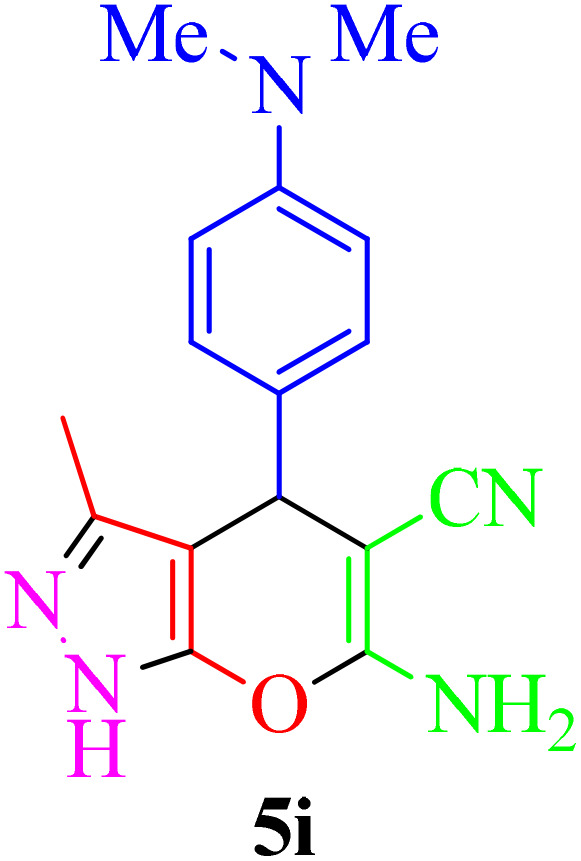	40	5.96 × 10^3^	2.38 × 10^5^	90	217–220	218–220 (ref. 35)
10	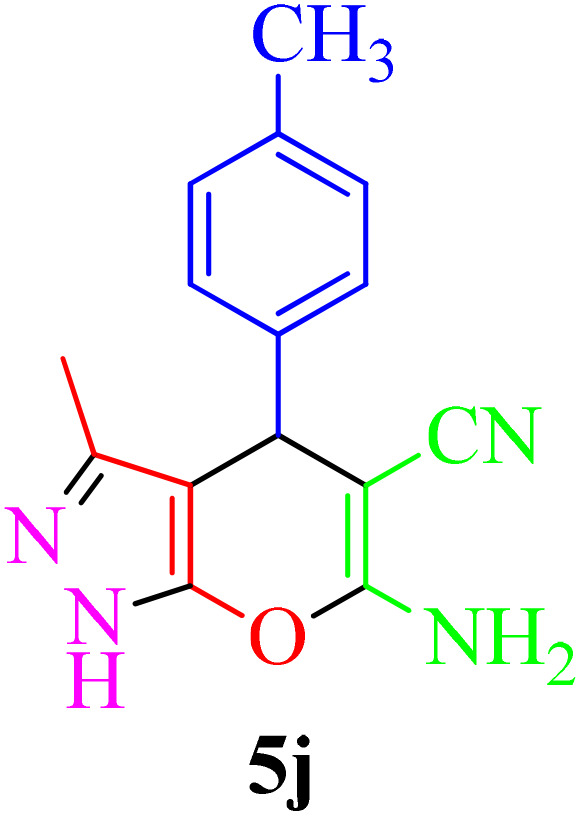	25	1.02 × 10^4^	2.54 × 10^5^	96	200–203	200–202 (ref. 29)
11	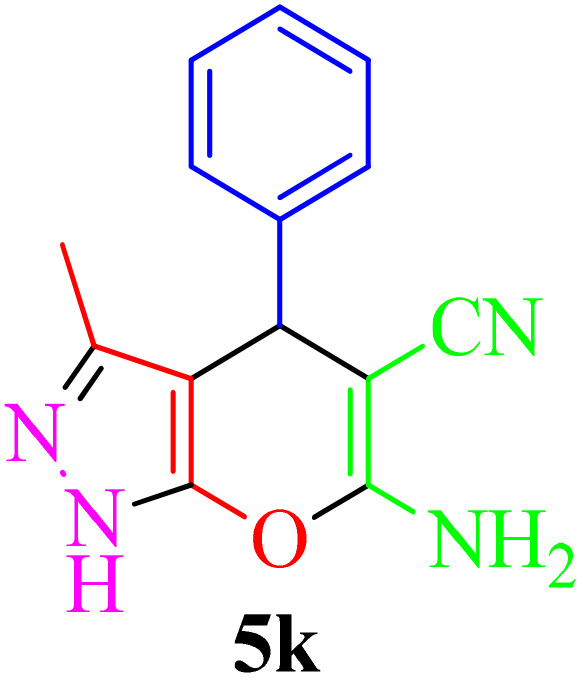	20	1.28 × 10^4^	2.57 × 10^5^	97	241–243	241–243 (ref. 29)

a Reaction conditions: aldehydes 5a–5k (1 mmol), malononitrile (1 mmol), ethyl acetoacetate (1 mmol), hydrazine hydrate (1 mmol), catalyst Re-NA–CH_2_CO_2_H (10 mg) in H_2_O at 25 °C.

b Isolated yield.

In catalyst-related studies, two key metrics are often discussed: Turnover Number (TON) and Turnover Frequency (TOF). The TON indicates the total number of substrate molecules that can be transformed into product by each molecule of catalyst, usually expressed as the yield (the quantity of product produced) divided by the amount of catalyst, measured in moles. This is represented in eqn (1).1
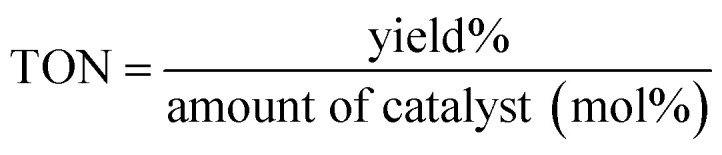


The TOF, on the other hand, measures how many substrate molecules a catalyst can convert into product per molecule of catalyst per unit of time. This is determined by dividing the Turnover Number (TON) by the duration of the reaction, as shown in eqn (2). A greater TOF value signifies a more effective catalyst, indicating that it can drive more reactions per active site in a given timeframe. Tables 2 and 4 display the TON and TOF values associated with pyrano[2,3-*c*]pyrazole and 2-amino-3 cyanopyridine derivatives.2
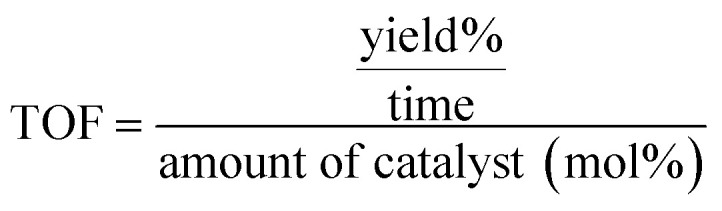


##### Proposed mechanism for the synthesis of pyrano[2,3-*c*]pyrazole

**Scheme 4:**
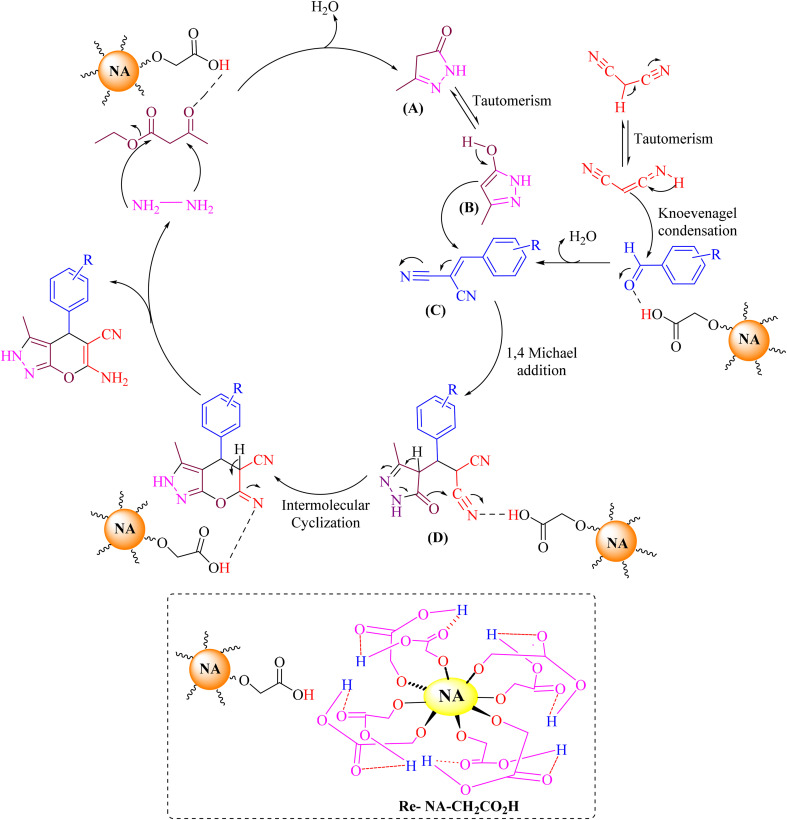
Proposed mechanism for the synthesis of pyrano[2,3-*c*]pyrazole with Re-NA–CH_2_CO_2_H catalyst.

The synthesis of pyrano[2,3-*c*]pyrazole in the presence of the catalyst Re-NA–CH_2_CO_2_H proceeds through a mechanistic pathway, as illustrated in Scheme 4. Initially, the carbonyl groups of ethyl acetoacetate are activated by the coordination and electron-withdrawing effect of the catalyst. This activation lowers the electron density at the carbonyl carbon, rendering it more electrophilic. Subsequently, hydrazine hydrate nucleophilically attacks the activated carbonyl group, leading to the formation of an intermediate pyrazolone (A). Intermediate A undergoes keto–enol tautomerization, resulting in the generation of its enolic tautomer (B). Simultaneously, the Knoevenagel condensation reaction occurs between malononitrile and an aldehyde, which is also activated by the catalyst. The activation of the aldehyde enhances its electrophilicity, enabling the nucleophilic attack of malononitrile's doubly conjugated system. This condensation step produces the arylidene malononitrile intermediate (C). In the subsequent step, the enolic tautomer (B) reacts with the arylidene malononitrile intermediate (C) *via* a Michael addition. This process involves the nucleophilic attack of the enolic oxygen or nitrogen on the α,β-unsaturated nitrile moiety of intermediate C, forming an adduct (D). The resultant intermediate D undergoes an intermolecular cyclization reaction, facilitated by the proximity of reactive functionalities and the catalytic influence of Re-NA–CH_2_CO_2_H. Finally, the cyclized intermediate undergoes further tautomerization, which redistributes the electronic structure and results in the formation of the final pyrano[2,3-*c*]pyrazole products. This mechanism highlights the critical role of the Re-NA–CH_2_CO_2_H catalyst in activating both substrates and promoting the sequential reactions that lead to the formation of the desired heterocyclic product.^36^

#### 3.2.2. Synthesis of the four-component one-pot 2-amino-3-cyanopyridine derivatives

**Scheme 5:**
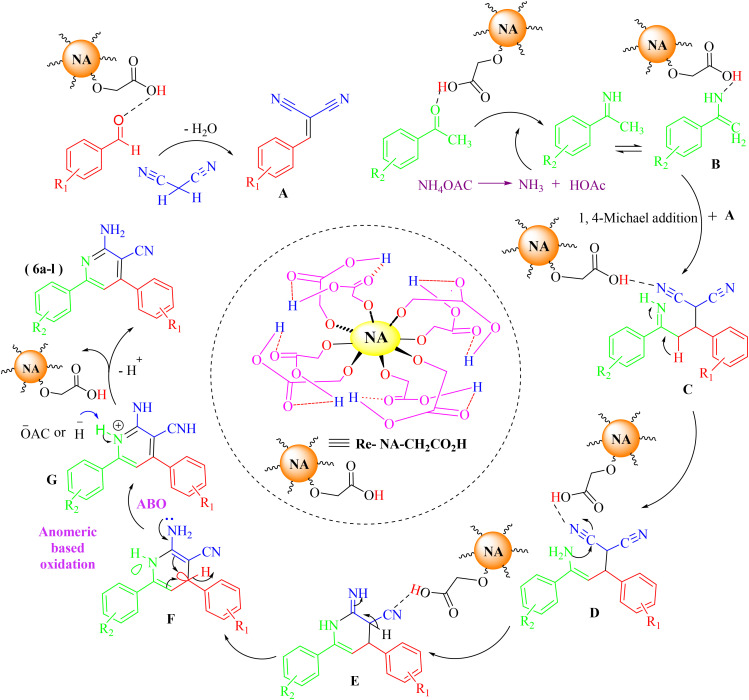
Proposed mechanism synthesis of 2-amino-3 cyanopyridine with Re-NA–CH_2_CO_2_H catalyst.

After identifying the Brønsted acid catalyst Re-NA–CH_2_CO_2_H, its catalytic performance was investigated in the synthesis of 2-amino-3-cyanopyridines *via* a vinylogous anomeric-based oxidation process. For achieving this goal, initially the four-component reaction of benzaldehyde, acetophenone, malononitrile and ammonium acetate was chosen as a model reaction (Scheme 5 and Table 3). After that, in order to find the best results in terms of yield and reaction time, we investigated the role of effective parameters such as solvents, the amount of catalyst Re-NA–CH_2_CO_2_H, temperature and reaction time. The achieved data indicated that the best results were obtained when 15 mg of catalyst Re-NA–CH_2_CO_2_H was utilized at 70 °C in H_2_O as solvent (Table 3, entry 10). In the following, to further investigate the activity of the synthesized catalyst Re-NA–CH_2_CO_2_H, other 2-amino-3 cyanopyridine derivatives were synthesized using different aldehydes and ketones. The results of these reactions are shown in Table 4.

**Table 3:** Optimization synthesis of 2-amino-3-cyanopyridine in the presence of Re-NA–CH_2_CO_2_H^a^

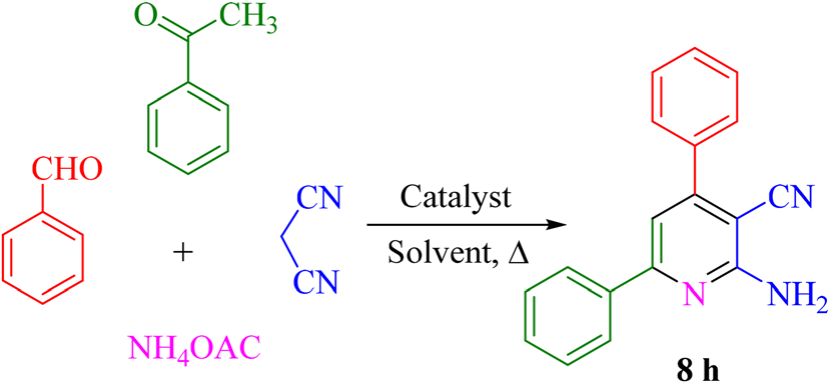				
Entry	Catalyst (mg)	Solvent	Temperature (°C)	Yield^b^ (%)
1	30	Water	90	98
2	30	Ethanol	Reflux	85
3	30	Water : ethanol (1 : 1)	Reflux	89
4	30	Ethyl acetate	Reflux	86
5	30	Dimethylformamide	90	88
6	30	*n*-Hexane	Reflux	35
7	20	Water	90	96
8	15	Water	90	96
9	10	Water	90	92
**10**	**15**	**Water**	**70**	**96**
11^c^	15	Water	70	N.R.^c^
12^d^	15	Water	70	25^d^
13	15	Water	50	89
14	—	Water	70	N.R.

a Reaction conditions: a mixture of benzaldehyde (1 mmol), acetophenone (1 mmol), malononitrile (1 mmol), ammonium acetate (1 mmol) and Re-NA–CH_2_CO_2_H (15 mg), solvent (3 mL) and 30 min.

b Isolated yield.

c Catalyst NA (15 mg), 4 h.

d Catalyst NA-oxide (15 mg), 30 min.

**Table 4:** Synthesis of 2-amino-3-cyanopyridine in the presence of Re-NA–CH_2_CO_2_H^a^

Entry	Product	Time (min)	TOF (min^−1^)	TON	Yield^b^ (%)	Mp (°C)	Mp (°C) ref.
1	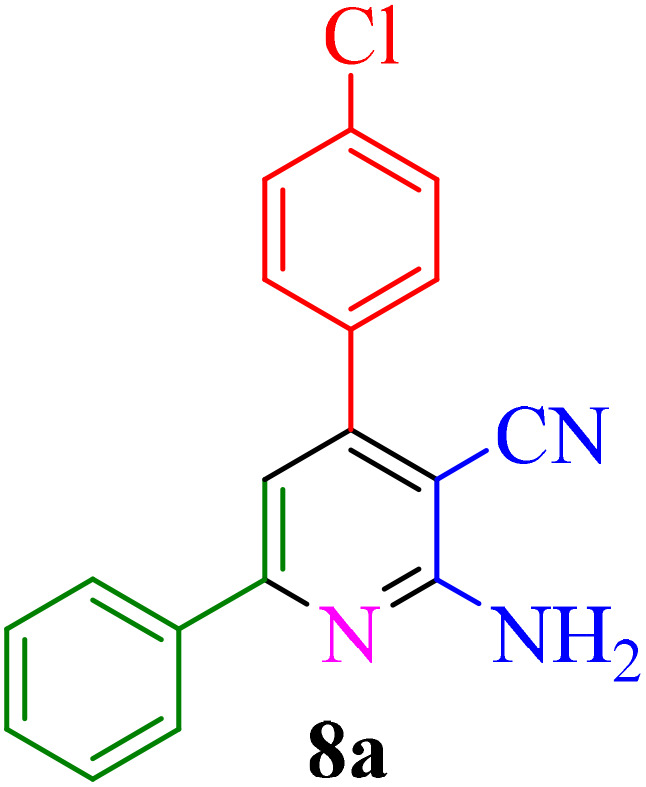	35	7.29 × 10^3^	2.55 × 10^5^	97	168–170	170–172 (ref. 38)
2	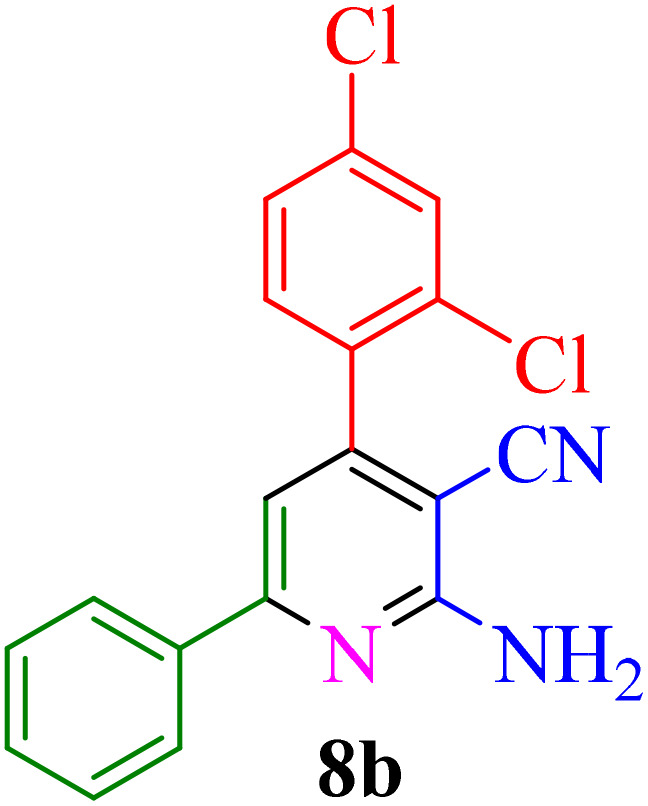	50	4.74 × 10^3^	2.37 × 10^5^	90	237–239	239–241 (ref. 38)
3	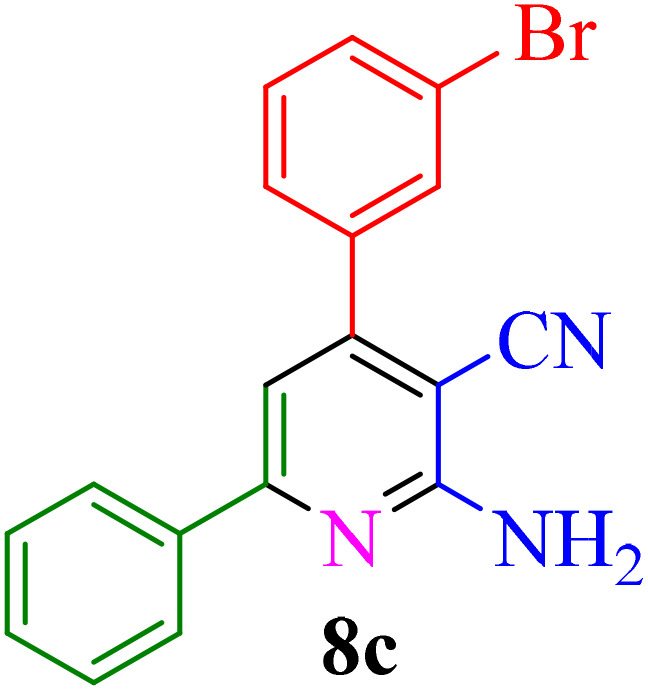	35	1.02 × 10^4^	2.42 × 10^5^	92	160–163	164–166 (ref. 39)
4	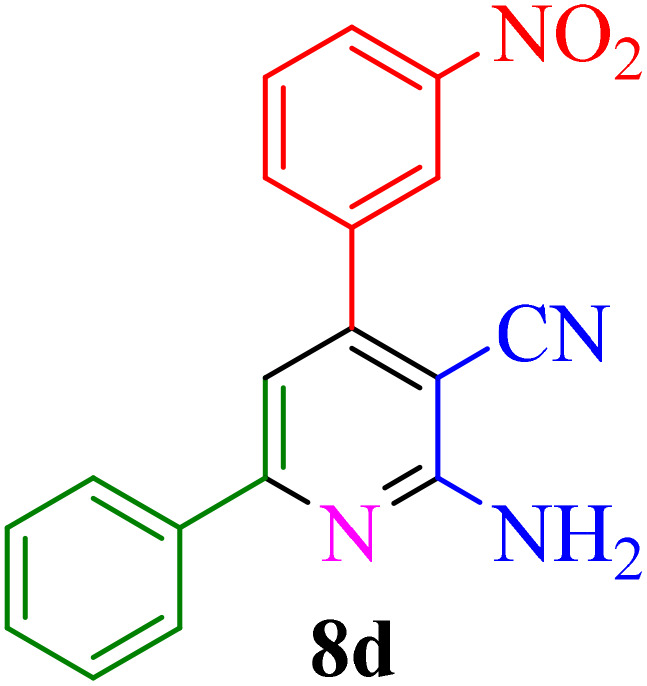	30	8.07 × 10^3^	2.42 × 10^5^	92	197–199	198–200 (ref. 40)
5	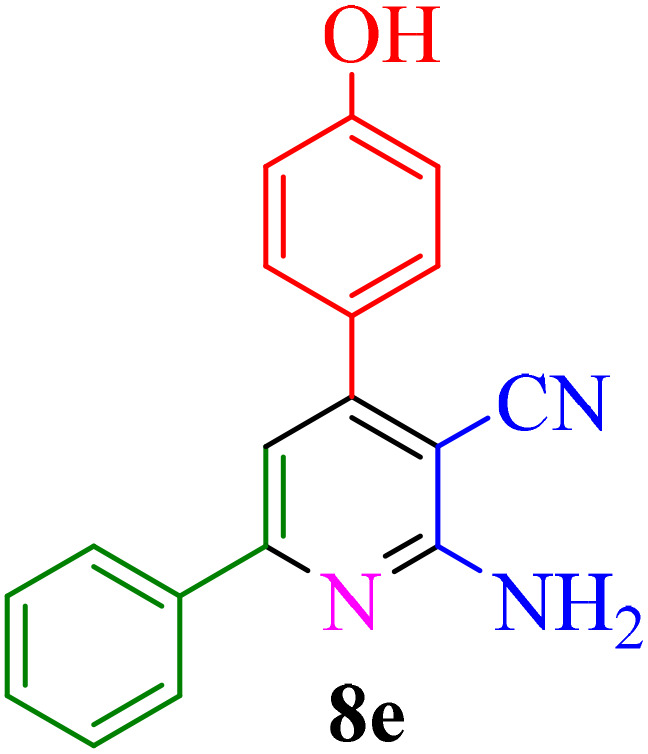	35	7.1 × 10^3^	2.47 × 10^5^	94	218–220	219–221 (ref. 41)
6	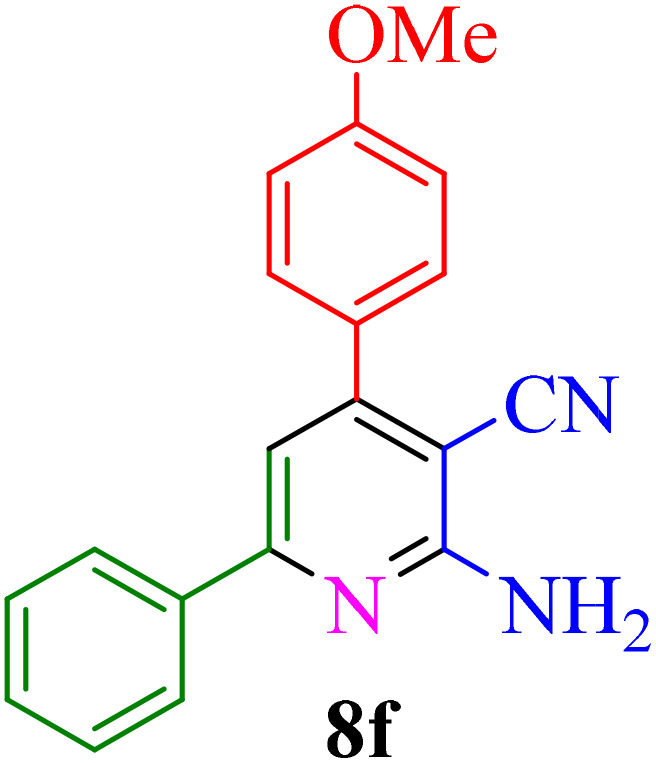	35	6.99 × 10^3^	2.44 × 10^5^	93	173–175	173–174 (ref. 42)
7	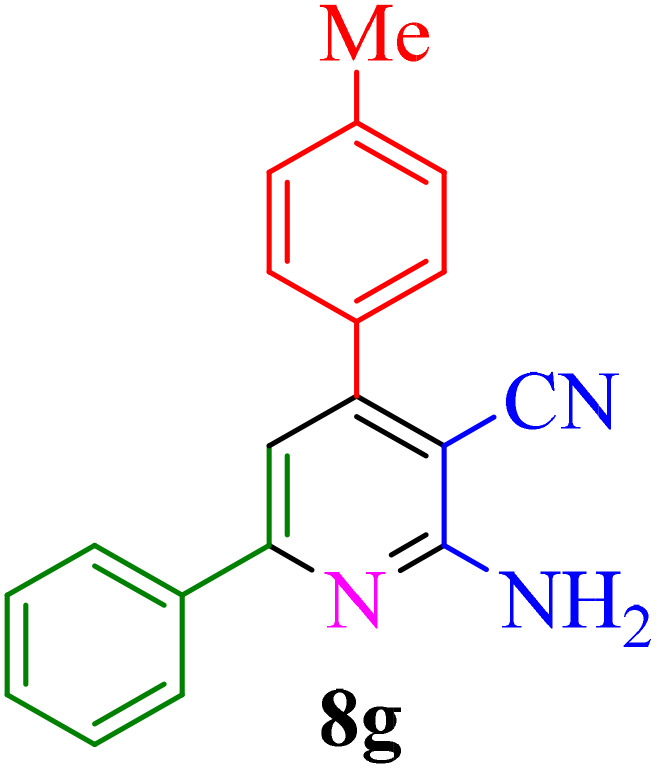	40	5.99 × 10^3^	2.39× 10^5^	91	173–175	173–174 (ref. 43)
8	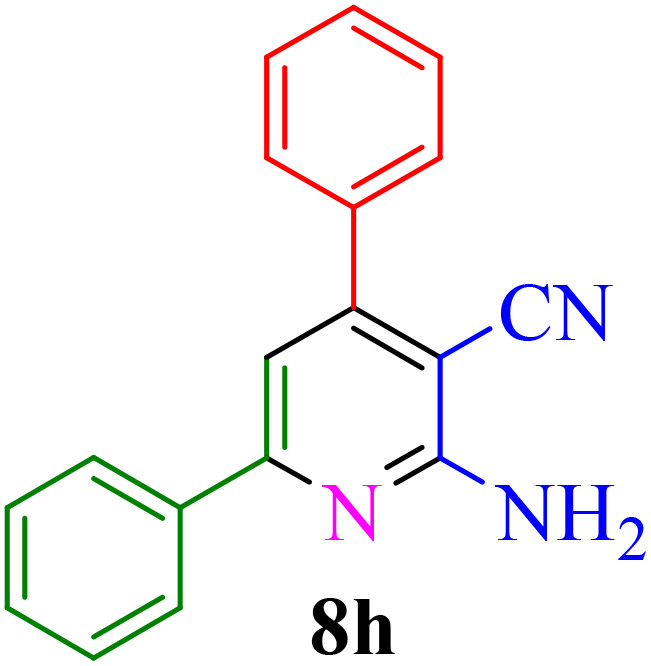	30	8.42 × 10^3^	2.53 × 10^5^	96	183–185	182–184 (ref. 38)
9	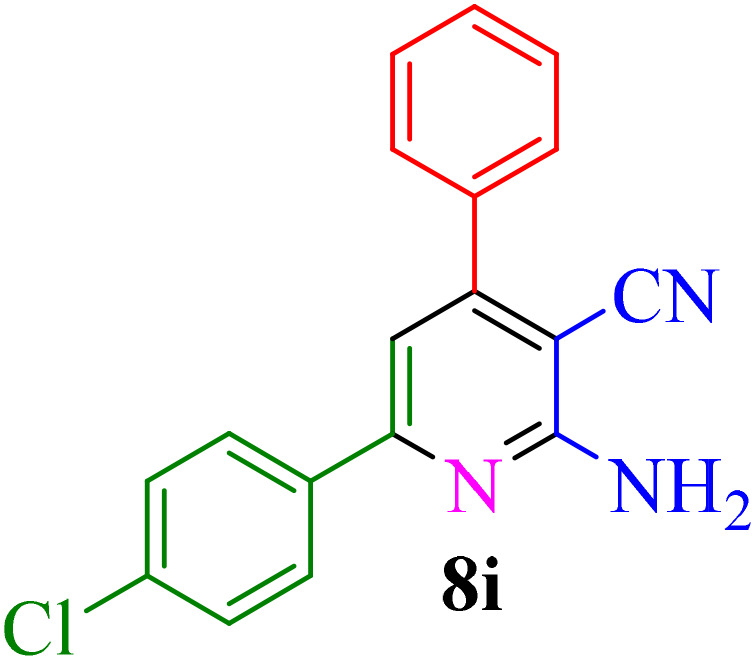	40	6.2 × 10^3^	2.5 × 10^5^	95	236–238	238–240 (ref. 44)
10	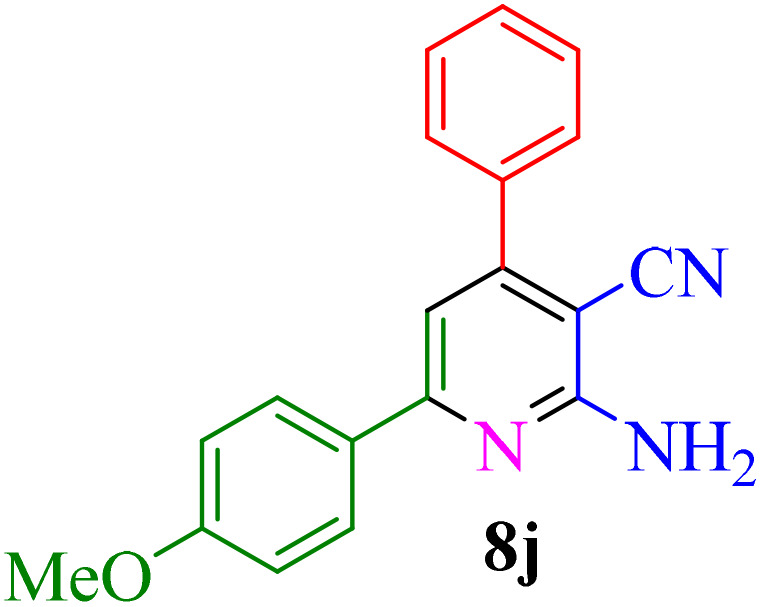	45	5.50 × 10^3^	2.47 × 10^5^	94	168–170	168–170 (ref. 41)
11	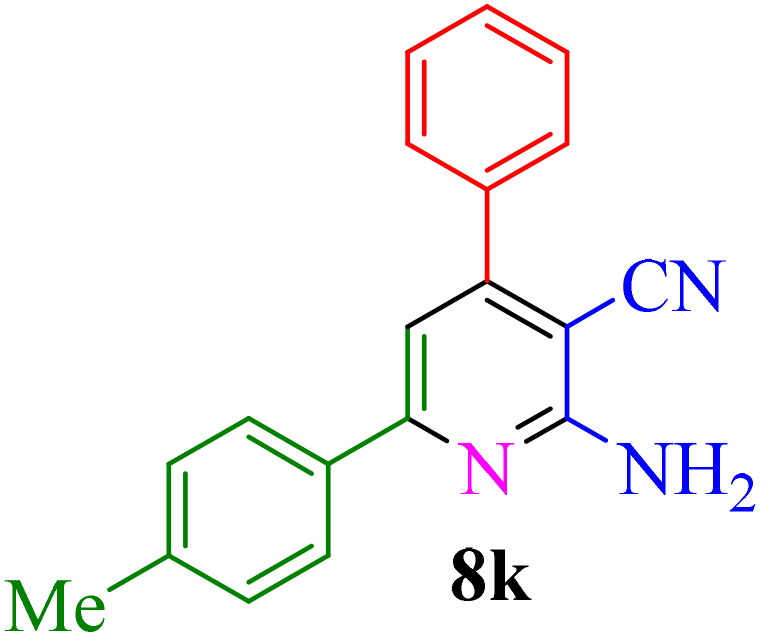	40	6.12 × 10^3^	2.45 × 10^5^	93	161–164	160–163 (ref. 45)
12	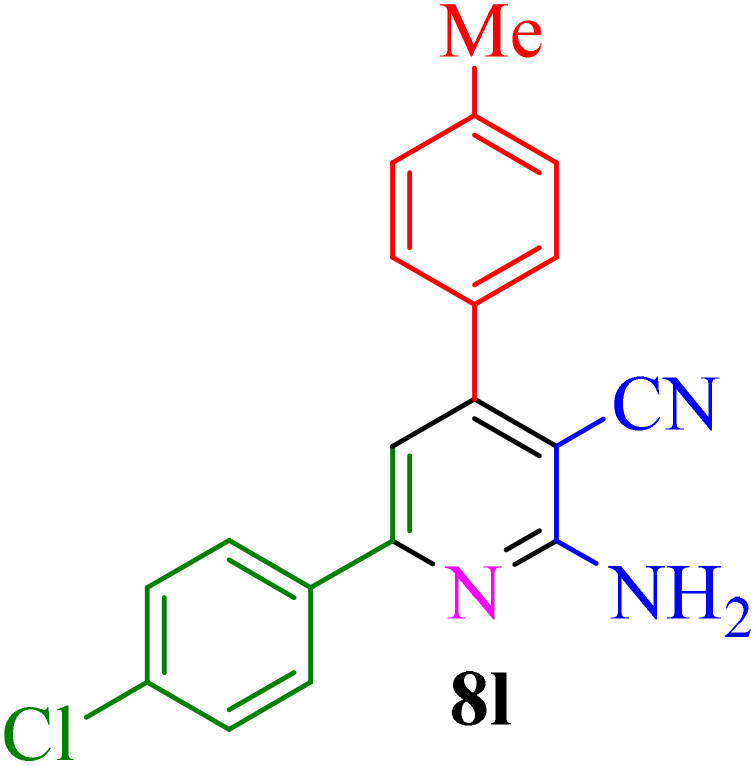	45	5.50 × 10^3^	2.47 × 10^5^	94	215–218	216–218 (ref. 46)

a Reaction conditions: aldehydes 8a–8l (1 mmol), acetophenone derivatives (1 mmol) malononitrile (1 mmol), ammonium acetate (1 mmol) and Re-NA–CH_2_CO_2_H (15 mg) in H_2_O at 70 °C.

b Isolated yield.

In addition to the solubility of reactants and products, solvents can also affect reaction rates, pathways, selectivity, and yields. Thus, for reactions such as the synthesis of pyrano[2,3-*c*]pyrazole derivatives and 2-amino-3-cyanopyridine, solvents play a key role. Water, as a protic and polar solvent, stabilizes intermediates through hydrogen bonding, potentially lowering activation energy barriers and increasing reaction rates. “Chemistry on water” has shown that the use of an aqueous medium can sometimes improve reaction rates and yields. Water, as a solvent, can also affect the acidity and availability of protons from Brønsted acid catalysts. This effect can lead to an increase in the rate of the synthesis of pyrano[2,3-*c*]pyrazole and 2-amino-3-cyanopyridine by facilitating the protonation of nucleophiles or activating electrophilic centers.^37^

##### Proposed mechanism for the synthesis of 2-amino-3 cyanopyridines

The proposed mechanism catalyzed by Re-NA–CH_2_CO_2_H for the synthesis of 2-amino-3-cyanopyridine is shown in Scheme 5. First, the catalyst activates the carbonyl group of aldehydes and ketones *via* protonation, generating highly reactive electrophiles. Next, the Knoevenagel condensation between the activated aldehyde and malononitrile occurs, producing the arylidene malononitrile intermediate A. Simultaneously, ammonium acetate reacts with the activated acetophenone to form the enamine intermediate B. In the subsequent step, the arylidene malononitrile intermediate A undergoes a Michael addition reaction with the enamine intermediate B, forming intermediate C. This step involves nucleophilic attack by the enamine on the electron-deficient double bond of the arylidene malononitrile. Intermediate C then undergoes sequential intramolecular cyclization, isomerization, and aromatization processes to generate intermediate F, which possesses a structure suitable for vinylogous anomeric-based oxidation. In this intermediate, both the endocyclic and exocyclic nitrogen atoms facilitate hydride removal, promoting the formation of the aromatic system. Finally, the products (8a–l) are obtained through deprotonation of intermediate G, completing the synthesis of the desired 2-amino-3-cyanopyridine compounds. This mechanism highlights the critical role of the Re-NA–CH_2_CO_2_H catalyst in activating substrates, facilitating key transformations, and driving the reaction toward the formation of the final aromatic products.^38,47^

### 3.3. Effect of pH

**Fig. 7:**
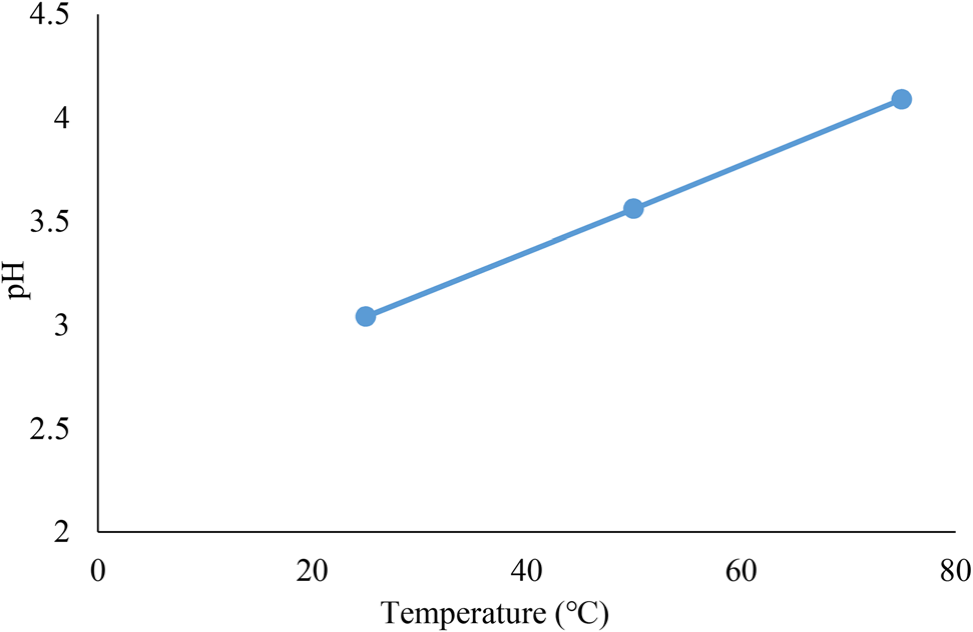
Measurement of pH of Brønsted acid catalyst Re-NA–CH_2_CO_2_H at 25, 50 and 70 °C.

For this objective, three different temperatures (25, 50, and 70 °C) were assessed to determine the acidity of the Re-NA–CH_2_CO_2_H catalyst with a glass electrode. Initially, 10 mg of the catalyst was mixed with 3 mL of deionized water at 25, 50, and 70 °C. The electrode indicated pH values of 3.04, 3.56, and 4.09, respectively (Fig. 7). The findings demonstrated that temperature had an effect on the pH level.

### 3.4. Reusability of the Re-NA–CH_2_CO_2_H

The recycling and reuse of the catalyst are highly important not only because they enhance economic viability but also because recyclability is one of the key principles of green chemistry. To assess the recyclability and reusability of the Re-NA–CH_2_CO_2_H catalyst, model reactions (5k and 8h) were conducted. After each reaction cycle, the catalyst was separated from the reaction mixture using filter paper, thoroughly washed with ethanol to remove any residual reactants or products, and dried at room temperature before being reused in subsequent cycles.

**Fig. 8:**
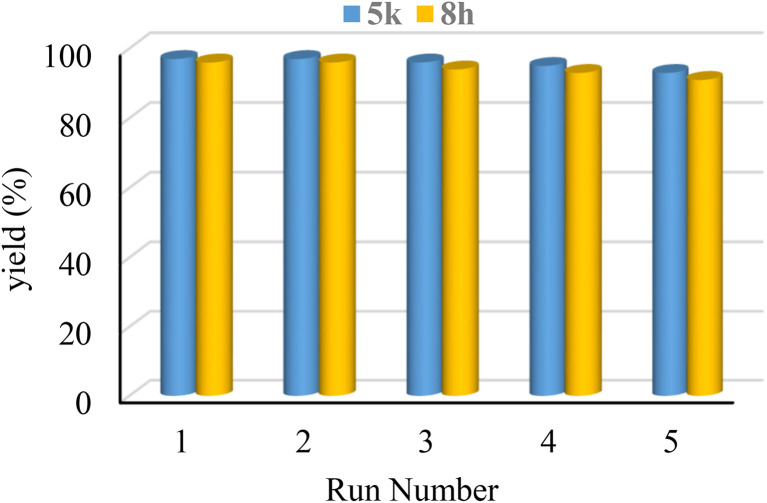
Reusability of Brønsted acid catalyst Re-NA–CH_2_CO_2_H in the synthesis of 5k and 8h products.

The results presented in Fig. 8 demonstrate that the Re-NA–CH_2_CO_2_H catalyst can be recovered and reused up to five times without significant loss of activity. This highlights the robustness and stability of the catalyst under reaction conditions, which aligns well with the principles of green chemistry by minimizing waste generation and reducing the need for fresh catalyst synthesis.

**Fig. 9:**
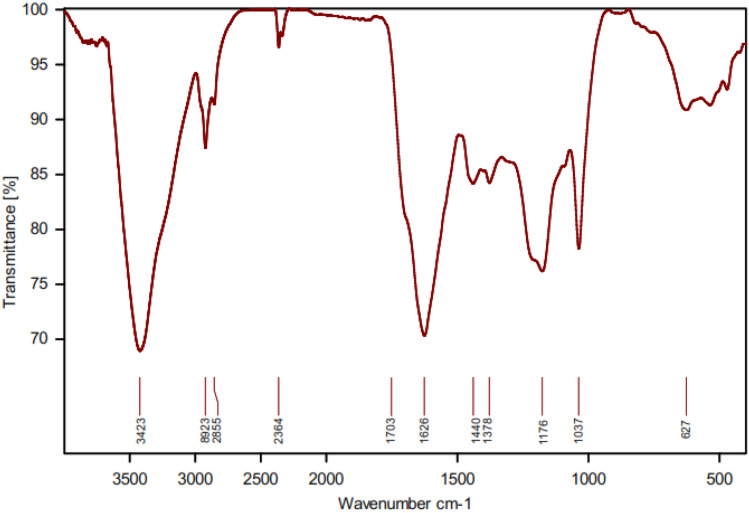
FT-IR spectra of after 5 steps recovery Brønsted acid catalyst Re-NA–CH_2_CO_2_H.

To further confirm the structural integrity of the catalyst after multiple cycles, an FT-IR spectrum was recorded for the recycled catalyst. As shown in Fig. 9, the FT-IR spectrum of the recovered catalyst closely matches that of the freshly synthesized material, indicating that the catalyst retains its functional groups and stability even after five stages of recycling and reuse. This finding underscores the durability and reusability of Re-NA–CH_2_CO_2_H, reinforcing its potential as a green and sustainable catalyst.

### 3.5. Hot filtration

The hot filtration test was conducted to ascertain the heterogeneous character of the Re-NA–CH_2_CO_2_H catalyst and assess potential leaching of acidic sites. Utilizing the reaction between 4-chlorobenzaldehyde, hydrazine hydrate, ethyl acetoacetate and malononitrile in water, the catalyst was removed after 10 minutes at 66% yield, followed by an additional 10 minutes of stirring. The resulting yield of 68% suggests that the acidic groups on the natural asphalt base are stable. This finding implies that there is minimal leaching of acidic groups into the solution or degradation of the catalyst.

### 3.6. Comparison of the catalytic activity of Re-NA–CH_2_CO_2_H with other catalysts

To demonstrate the superiority and high efficiency of the Brønsted acid catalyst Re-NA–CH_2_CO_2_H synthesized in this research, the results obtained for the synthesis of 6-amino-3-methyl-4-phenyl-1,4-dihydropyrano[2,3-*c*]pyrazole-5-carbonitrile (Table 5, entries 1–6) and 2-amino-4,6-diphenylnicotinonitrile (Table 5, entries 7–13) were compared with catalysts reported in previous studies. As shown in Table 5, this catalyst Re-NA–CH_2_CO_2_H has advantages such as mild reaction conditions, high catalytic activity, short reaction time, and high yield.

**Table 5:** Comparison of the catalytic activity of Brønsted acid catalyst Re-NA–CH_2_CO_2_H with other catalysts in the synthesis of products 5k (entries 1–6) and 8h (entries 7–13)

Entry	Catalyst	Experimental conditions	Time (min)	Yield (%)	Eff^a^
1	Ag/TiO_2_ nano-thin films	H_2_O : EtOH (1 : 2 mL), 70 °C	30	88 (ref. 48)	
2	Nano-SiO_2_ (10 mol%)	H_2_O, 80 °C	30	94 (ref. 49)	
3	Fe_3_O_4_@THAM–SO_3_H	Ethanol : H_2_O, 100 °C	5–25	82 (ref. 50)	
4	Borax (0.04 g)	H_2_O, reflux	50	85 (ref. 30)	21.25
5	FSiPSS nano-catalyst (20 mg)	EtOH, 40 °C, ultrasonic		80 (ref. 51)	40
**6**	**Re-NA–CH**_**2**_ **CO**_**2**_ **H (10 mg)**	**H**_**2**_ **O, 25°C**	**20**	**97 (This work)**	**97**
7	Iron(iii) phosphate (10 mol%)	EtOH, reflux	240	82 (ref. 52)	
8	PDMAF-MNPs (40 mg)	EtOH (reflux)	150	92 (ref. 53)	23
9	GO (10 mol%)	H_2_O, 80 °C	300	96 (ref. 54)	
10	Na_2_CaP_2_O_7_ (0.05 g)	Solvent-free, 80 °C	30	92 (ref. 55)	18.4
11	LDH@TRMS@NH_2_SO_2_(C_2_H_4_)SO_2_NH_2_@nano copper (50 mg)	Solvent-free, 60 °C	12	88 (ref. 38)	17.6
12	Nanomagnetic catalyst bearing morpholine tags (14 mg)	Solvent-free, 80 °C	20	85 (ref. 46)	60.71
**13**	**Re-NA–CH**_**2**_ **CO**_**2**_ **H (15 mg)**	**H**_**2**_ **O, 70°C**	**30**	**96 (This work)**	**64**

a Eff = mmol of product formed per g of catalyst.

## 4. Conclusions

In this study, a novel heterogeneous Brønsted acid catalyst Re-NA–CH_2_CO_2_H was successfully synthesized using natural asphalt, an economical, readily available, biocompatible, and recyclable carbon substrate. The catalyst was comprehensively characterized using advanced techniques such as FT-IR, TGA, SEM, EDX, and TEM, confirming its structural integrity and functionality.

The catalytic activity of Re-NA–CH_2_CO_2_H was evaluated in the synthesis of multicomponent reactions for the production of pyrano[2,3-*c*]pyrazole and 2-amino-3-cyanopyridine derivatives. The results demonstrated that the catalyst exhibits high efficiency, selectivity, and stability under mild reaction conditions, making it suitable for green chemistry applications. The catalyst showed excellent recyclability, maintaining its catalytic activity over five consecutive cycles without significant degradation. This highlights its potential for sustainable and cost-effective use in industrial processes. Water was employed as the reaction solvent, emphasizing the environmentally friendly nature of the methodology. Water's advantages include its wide availability, non-toxicity, non-flammability, and ability to enhance reactivity and selectivity compared to conventional organic solvents. The catalyst facilitated the synthesis of pyrano[2,3-*c*]pyrazole and 2-amino-3-cyanopyridine derivatives with high yields and short reaction times, even at low loadings (10–15 mg). These heterocyclic compounds possess significant pharmaceutical and biological activities, underscoring the practical importance of the developed methodology.

Overall, the successful synthesis of Re-NA–CH_2_CO_2_H demonstrates the versatility and effectiveness of natural asphalt as a renewable carbon source for preparing functionalized Brønsted acid catalysts. This work aligns with the principles of green chemistry by promoting sustainable practices, reducing waste, and minimizing environmental impact. The findings suggest that Re-NA–CH_2_CO_2_H is a promising candidate for the efficient and eco-friendly synthesis of pharmaceutically relevant heterocyclic compounds.

## Author contributions

Shabnam Rashidi: validation, methodology, investigation, writing – original draft, conceptualization. Mohammad Soleiman-Beigi: funding acquisition, supervision, project administration, conceptualization, editing, resources.

## Conflicts of interest

All authors declare that there are no competing interests.

## Supplementary Material

RA-015-D5RA03786G-s001

## Data Availability

The data that support the findings of this study are available in ESI.†
