# Supplementary material for: Natural asphalt oxide-grafted carboxylic acid: a sustainable heterogeneous catalyst for synthesis of pyrano[2,3-c]pyrazoles and 2-amino-3-cyanopyridines in water
Source: RSC Adv. 2025 Jul 16;15(31):25274–90. doi: 10.1039/d5ra03786g (PMC12265887; doi:10.1039/d5ra03786g)
Supplement: RA-015-D5RA03786G-s001 [file RA-015-D5RA03786G-s001.pdf]

Supplementary Information (SI) for RSC Advances.
This journal is © The Royal Society of Chemistry 2025

Supporting Information

Natural Asphalt Oxide-Grafted Carboxylic Acid: A Sustainable Heterogeneous Catalyst for Synthesis of Pyrano[2,3-c] pyrazoles and 2-Amino-3-cyanopyridines in water

Shabnam Rashidi[a], Mohammad Soleiman-Beigi*[a]

[a]*Department of Chemistry, Faculty of Basic Sciences, Ilam University, P.O. Box 69315-516, Ilam, Iran*

E-mail: SoleimanBeigi@yahoo.com & m.soleimanbeigi@ilam.ac.ir

TABLE OF CONTENTS

Contents **Page**

Figure S1. SEM image of the Brønsted acid nanocatalyst Re-NA-CH_2CO_2H …………………………………………...............S3

Figure S2. TEM image of the Brønsted acid nanocatalyst Re-NA-CH_2CO_2H……………………...…………….……….....S3

Figure S3. FT-IR spectra of 6-amino-4-(4-bromophenyl)-3-methyl-1,4-dihydropyrano[2,3-c] pyrazole-5-carbonitrile...............S4

Figure S4. ^{1}H NMR spectra of 6-amino-4-(4-bromophenyl)-3-methyl-1,4-dihydropyrano[2,3-c] pyrazole-5-carbonitrile…..…S5

Figure S5. ^{13}C NMR spectra of 6-amino-4-(4-bromophenyl)-3-methyl-1,4-dihydropyrano[2,3-c] pyrazole-5-carbonitrile.……S5

Figure S6. FT-IR spectra of 6-amino-3-methyl-4-(3-nitrophenyl)-1,4-dihydropyrano[2,3-c] pyrazole-5-carbonitrile..................S6

Figure S7. ^{1}H NMR spectra of 6-amino-3-methyl-4-(3-nitrophenyl)-1,4-dihydropyrano[2,3-c] pyrazole-5-carbonitrile.……....S7

Figure S8. ^{13}C NMR spectra of 6-amino-3-methyl-4-(3-nitrophenyl)-1,4-dihydropyrano[2,3-c] pyrazole-5-carbonitrile…..… S7

Figure S9. FT-IR spectra of 6-amino-3-methyl-4-phenyl-1,4-dihydropyrano[2,3-c] pyrazole-5-carbonitrile............................. S8

Figure S10. ^{1}H NMR spectra of 6-amino-3-methyl-4-phenyl-1,4-dihydropyrano[2,3-c] pyrazole-5-carbonitrile………….........S9

Figure S11. ^{13}C NMR spectra of 6-amino-3-methyl-4-phenyl-1,4-dihydropyrano[2,3-c] pyrazole-5-carbonitrile……….……...S9

Figure S12. FT-IR spectra of 2-amino-4-(4-chlorophenyl)-6-phenylnicotinonitrile...S10

Figure S13. ^{1}H NMR spectra of 2-amino-4-(4-chlorophenyl)-6-phenylnicotinonitrile……………………………………..…S11

Figure S14. ^{13}C NMR spectra of 2-amino-4-(4-chlorophenyl)-6-phenylnicotinonitrile…………………………………….........S11

Figure S15. FT-IR spectra of 2-amino-4,6-diphenylnicotinonitrile…………………………………………………….............S12

Figure S16. ^{1}H NMR spectra of 2-amino-4,6-diphenylnicotinonitrile…………………………………………………..…......S13

Figure S17. ^{13}C NMR spectra of 2-amino-4,6-diphenylnicotinonitrile…………………………………………………..……S13

Figure S18. FT-IR spectra of 2-amino-6-(4-methoxyphenyl)-4-phenylnicotinonitrile..S14

Figure S19. ^{1}H NMR spectra of 2-amino-6-(4-methoxyphenyl)-4-phenylnicotinonitrile……………………………….…….S15

Figure S20. ^{13}C NMR spectra of 2-amino-6-(4-methoxyphenyl)-4-phenylnicotinonitrile……………………………….…..…..S15

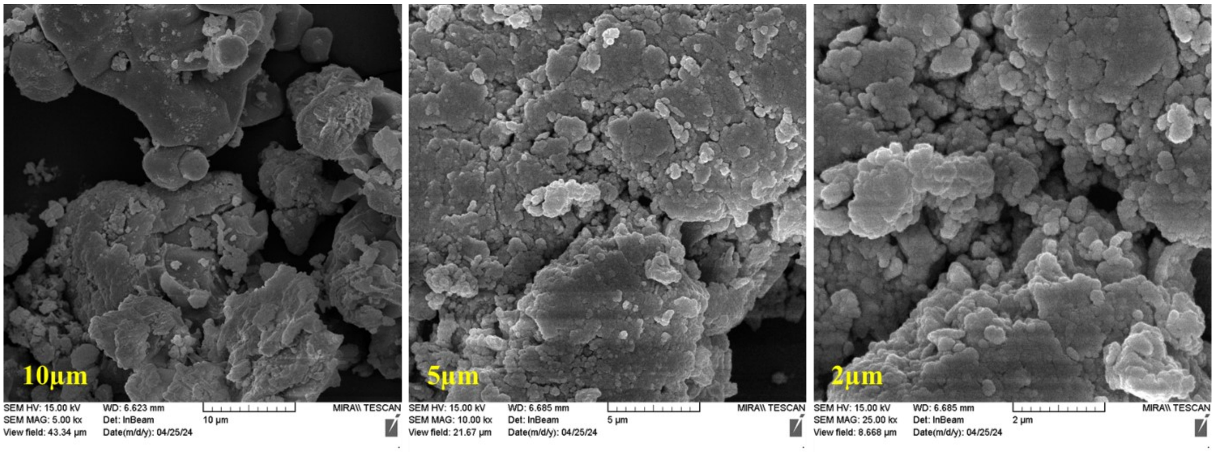

Figure S1. SEM image of the Brønsted acid nanocatalyst Re-NA-CH_2CO_2H

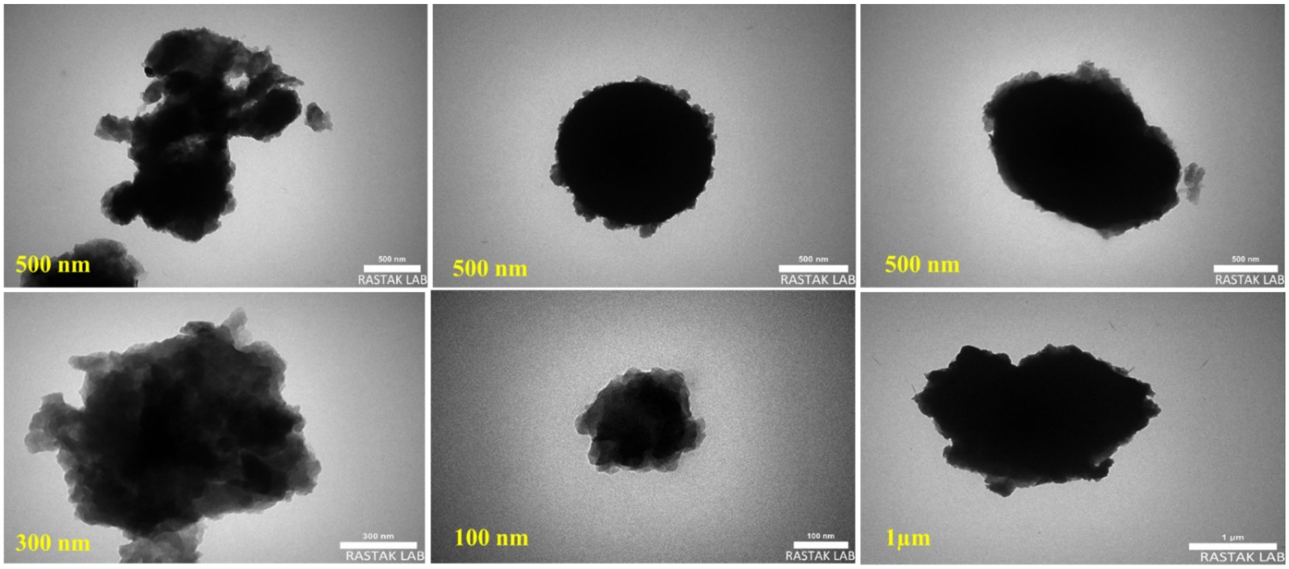

Figure S2. TEM image of the Brønsted acid nanocatalyst Re-NA-CH_2CO_2H

Spectroscopic data

All the products of the reactions of pyrano [2, 3-c] pyrazole and 2-amino-3 cyanopyridine described here are known compounds and the spectroscopic data were compared with the literature values. Data for some of the compounds are given below.

1: 6-amino-4-(4-bromophenyl)-3-methyl-1,4-dihydropyrano[2,3-c]pyrazole-5-carbonitrile (Table 2, 5c, Figure S3-Figure S5): Melting point: 178-180 °C. FT-IR (KBr) ν (cm^{-1}): 3392, 13182, 2189, 1643, 1601, 1489, 1402, 1010, 540 cm^{-1}. ^{1}H NMR (250 MHz, DMSO- d_6): δ (ppm)= 12.12 (s, 1H, NH), 7.10 – 7.50 (d-d, 4H), 6.91 (s, 2H, NH_2), 4.60 (s, 1H, CH), 1.77 (s, 3H, CH_3). ^{13}C NMR (63 MHz, DMSO-d_6): δ (ppm)= 155.66, 149.46, 138.64, 130.44, 126.12, 124.49, 115.39, 114.51, 91.87, 51.46, 4.49.

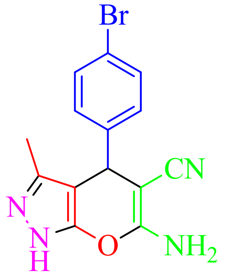

5c

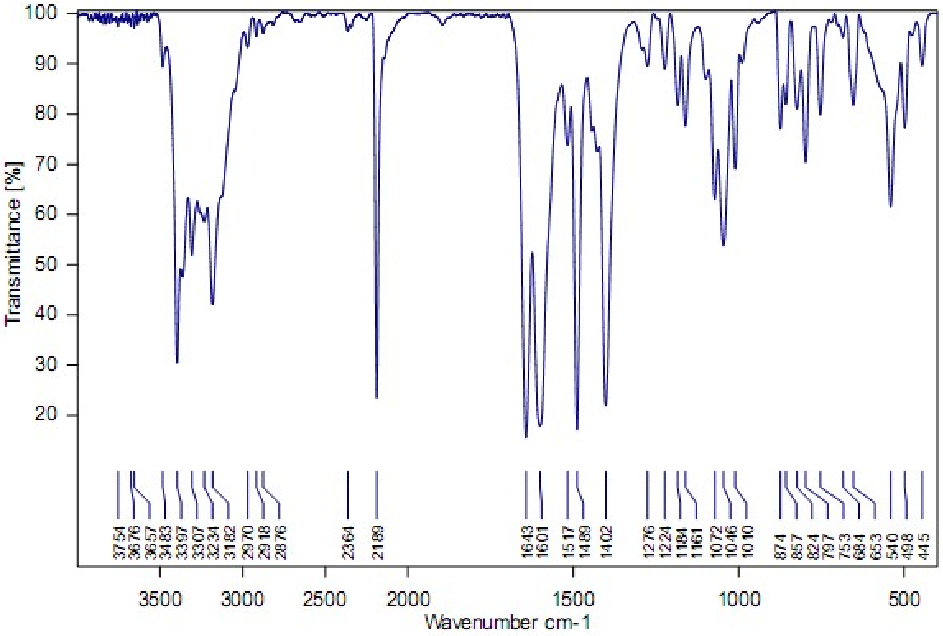

Figure S3. FT-IR spectra of 6-amino-4-(4-bromophenyl)-3-methyl-1,4-dihydropyrano[2,3-c]pyrazole-5-carbonitrile

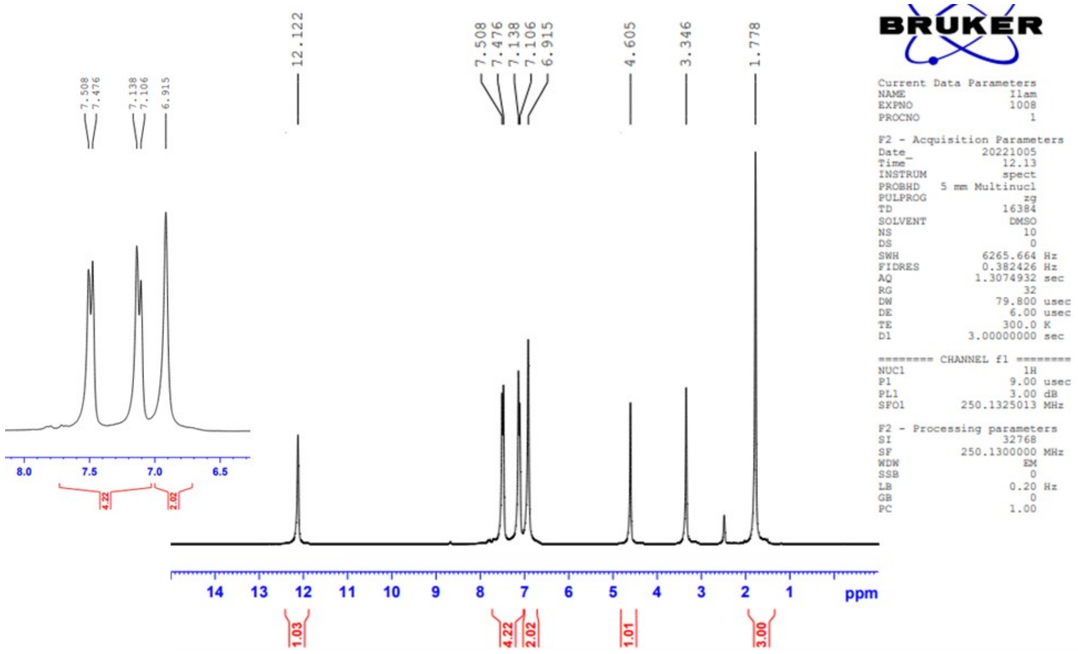

Figure S4. ^{1}HNMR spectra of 6-amino-4-(4-bromophenyl)-3-methyl-1,4-dihydropyrano[2,3-c]pyrazole-5-carbonitrile

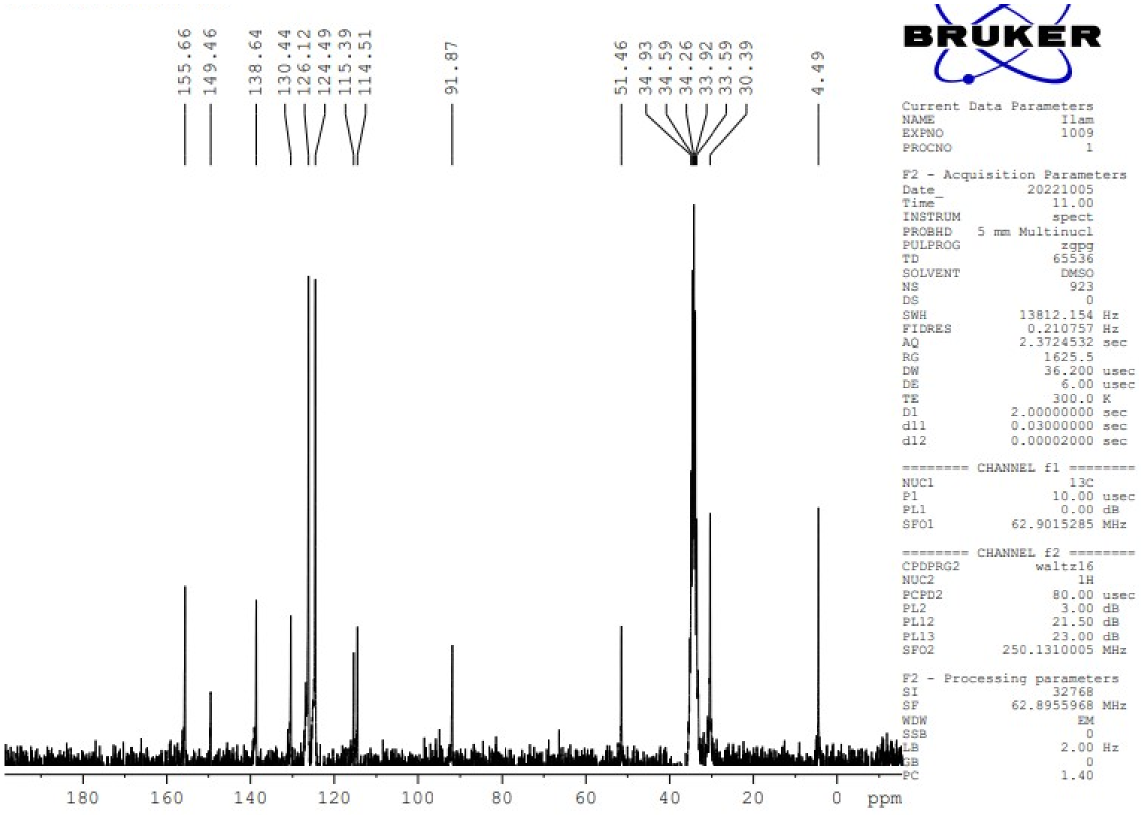

Figure S5. ^{13}CNMR spectra of 6-amino-4-(4-bromophenyl)-3-methyl-1,4-dihydropyrano[2,3-c]pyrazole-5-carbonitrile

2: 6-amino-3-methyl-4-(3-nitrophenyl)-1,4-dihydropyrano[2,3-c] pyrazole-5-carbonitrile

(Table 2, 5h, Figure S6-Figure S8): Melting point: 215-217 °C. FT-IR (KBr) ν (cm^{-1}): 3474, 3225, 2194, 1653, 1520cm^{-1}. ^{1}H NMR (250 MHz, DMSO- d_6): δ (ppm)= 12.57 (s, 1H, NH), 7.62 – 8.10 (m, 4H), 7.04 (s, 2H, NH_2), 4.74 (s, 1H, CH), 1.93 (s, 3H, CH_3). ^{13}C NMR (63 MHz, DMSO-d_6): δ (ppm)= 155.92, 149.47, 142.66, 141.58, 130.68, 129.14, 124.98, 116.73, 116.61, 115.27, 91.42, 50.94, 4.50.

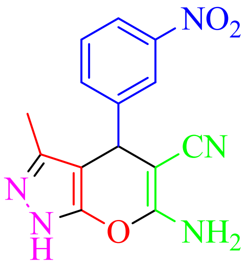

5h

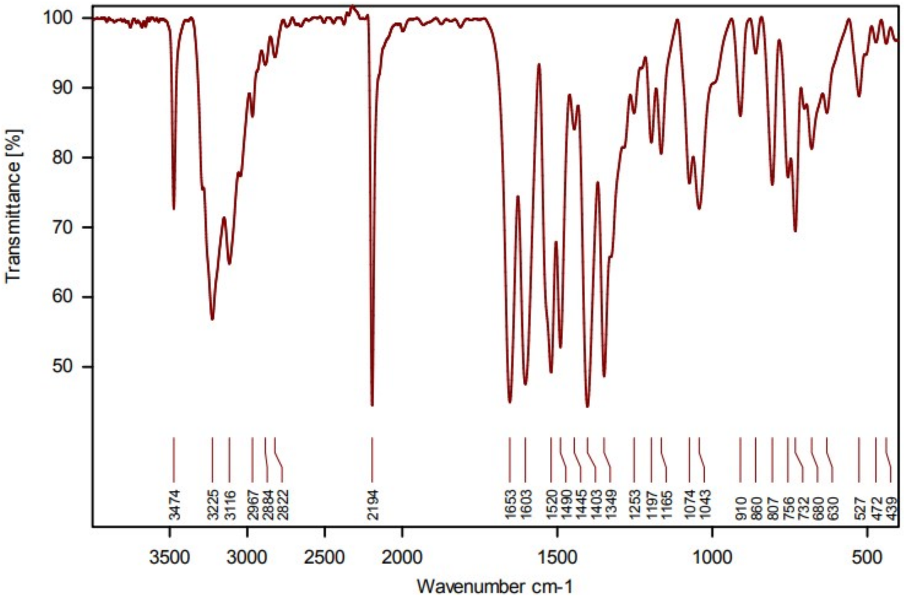

Figure S6. FT-IR spectra of 6-amino-3-methyl-4-(3-nitrophenyl)-1,4-dihydropyrano[2,3-c] pyrazole-5-carbonitrile

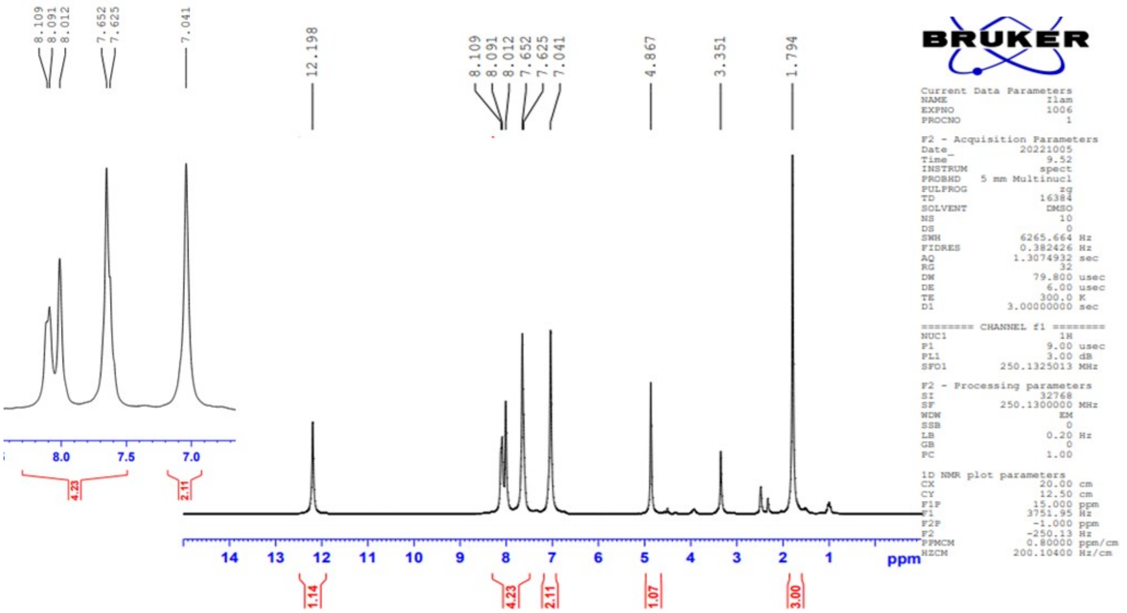

Figure S7. ^{1}HNMR spectra of 6-amino-3-methyl-4-(3-nitrophenyl)-1,4-dihydropyrano[2,3-c] pyrazole-5-carbonitrile

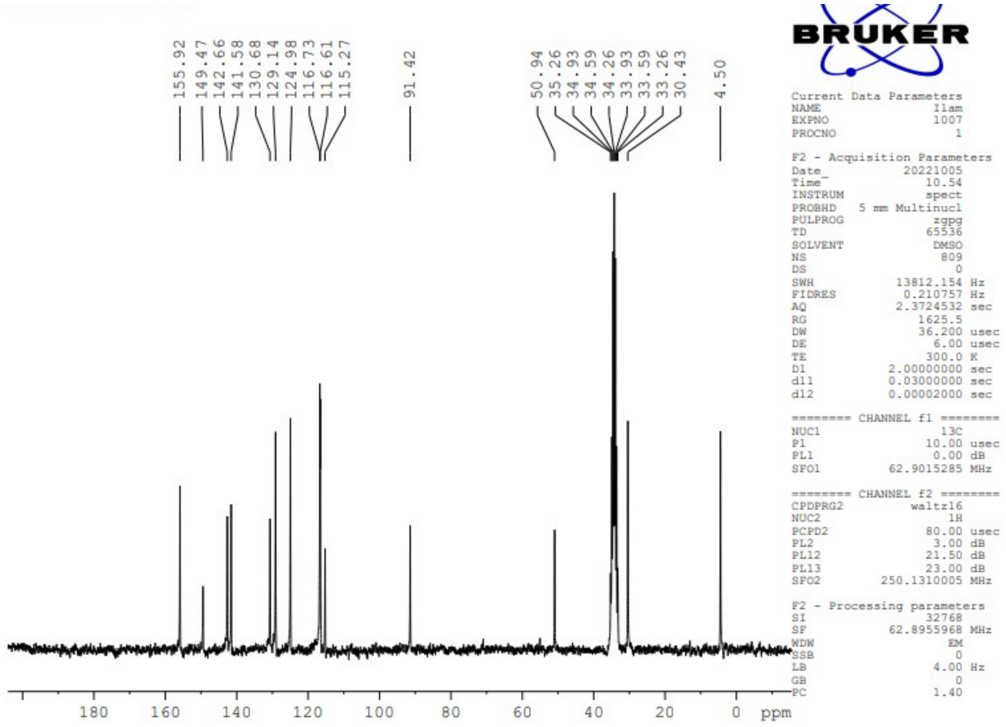

Figure S8. ^{13}CNMR spectra of 6-amino-3-methyl-4-(3-nitrophenyl)-1,4-dihydropyrano[2,3-c] pyrazole-5-carbonitrile

3: 6-amino-3-methyl-4-phenyl-1,4-dihydropyrano[2,3-c] pyrazole-5-carbonitrile (Table 2, 5k, Figure S9-Figure S11): Melting point: 241-243 °C. FT-IR (KBr) ν (cm^{-1}): 3374, 3023, 2192, 1649, 1517 cm^{-1}. ^1H NMR (250 MHz, DMSO- d_6): δ (ppm)= 12.07 (s, 1H, NH), 7.16– 7.29 (m, 5H), 6.85 (s, 2H, NH_2), 4.57 (s, 1H, CH), 1.76 (s, 3H, CH_3). ^{13}C NMR (63 MHz, DMSO-d_6): δ (ppm)= 155.64, 149.54, 138.64, 139.21, 130.34, 123.20, 122.23, 121.49, 115.56, 92.40, 51.96, 31, 4.49.

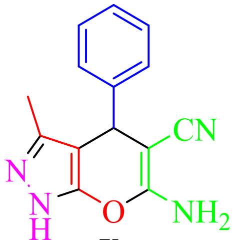

5k

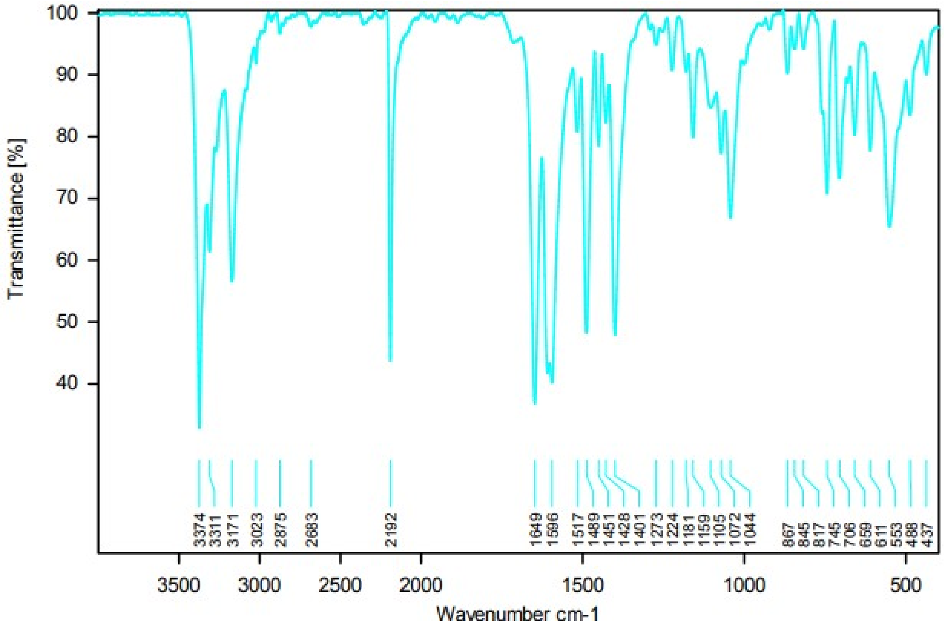

Figure S9. FT-IR spectra of 6-amino-3-methyl-4-phenyl-1,4-dihydropyrano[2,3-c] pyrazole-5-carbonitrile

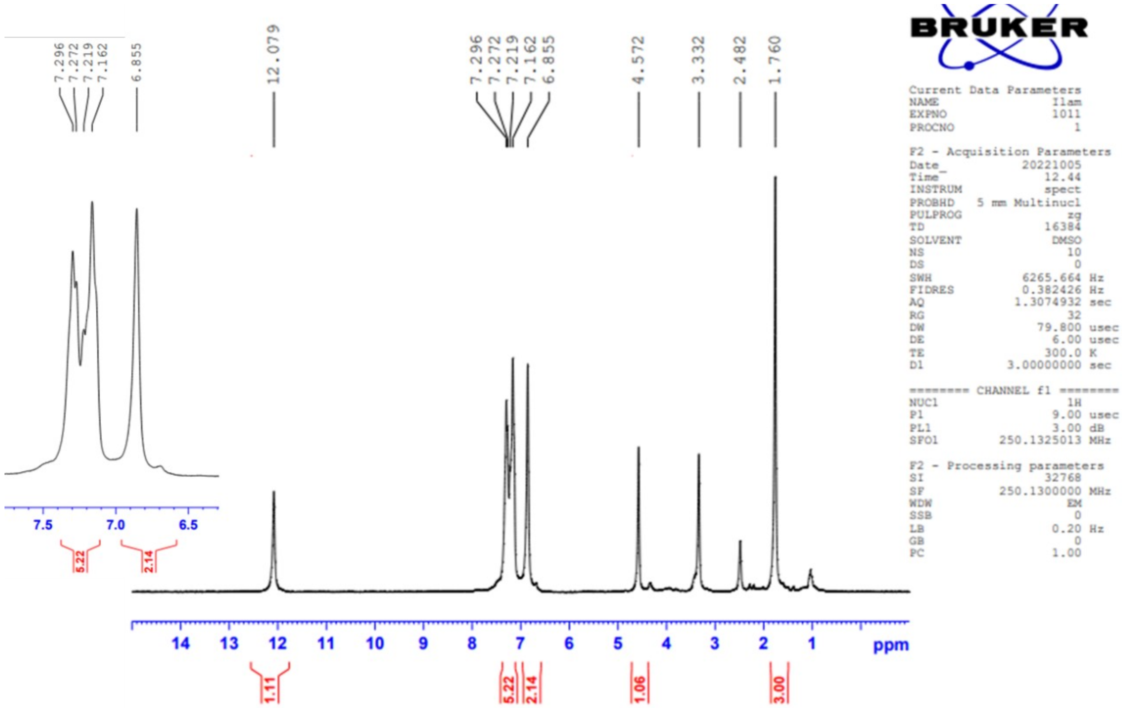

Figure S10. ^{1}HNMR spectra of 6-amino-3-methyl-4-phenyl-1,4-dihydropyrano[2,3-c] pyrazole-5-carbonitrile

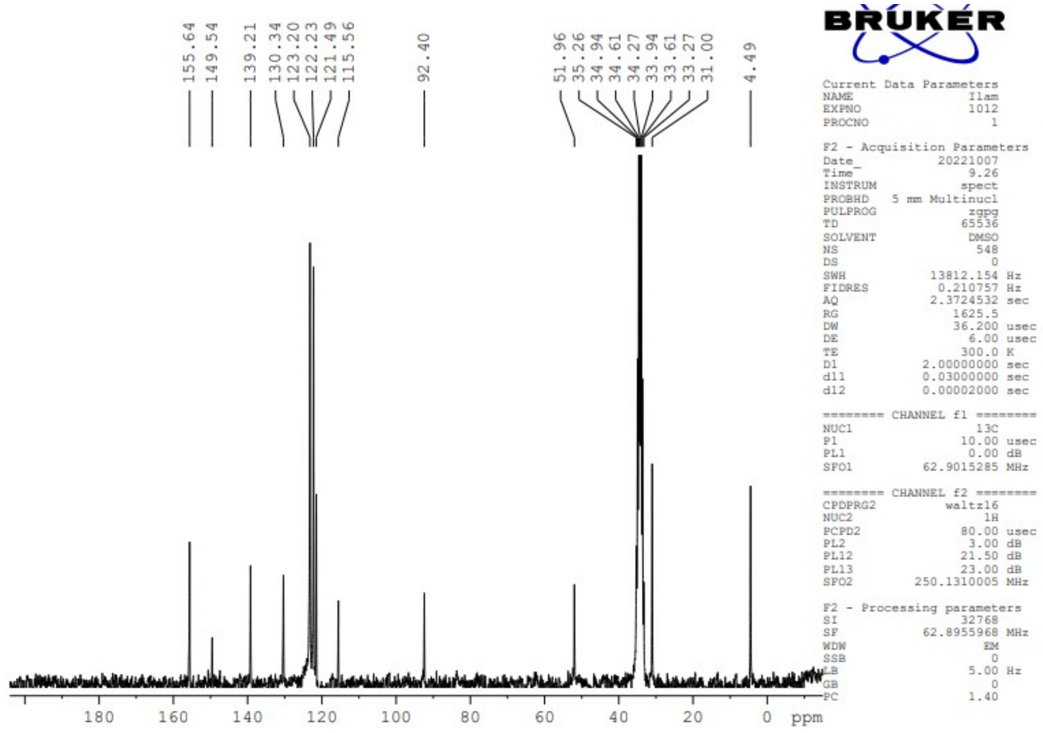

Figure S11. ^{13}CNMR spectra of 6-amino-3-methyl-4-phenyl-1,4-dihydropyrano[2,3-c] pyrazole-5-carbonitrile

4. 2-amino-4-(4-chlorophenyl)-6-phenylnicotinonitrile (Table 4, 6a, Figure S12-Figure S14): Melting point: 168-170 °C. FT-IR (KBr) ν (cm^{-1}): 3364, 3226, 3098, 2224, 1662, 1586, 1529, 1488, 1409, 1367, 1291, 1189, 1093, 827, 776, 704 and 658 cm^{-1}. ^{1}H NMR (250 MHz, DMSO-d_6): δ (ppm)= 8.51 (s, 2H, NH_2), 7.29-7.93 (m, 10 H). ^{13}C NMR (63 MHz, DMSO-d_6): δ (ppm)= 158.99, 155.56, 154.86, 138.01, 133.81,128.12, 126.89, 124.82, 124.47, 123.90, 123.31, 120.72, 108.79, 107.75, 76.99.

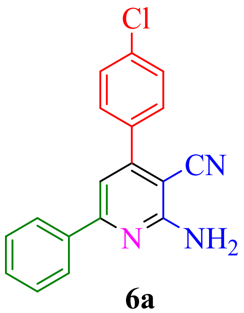

6a

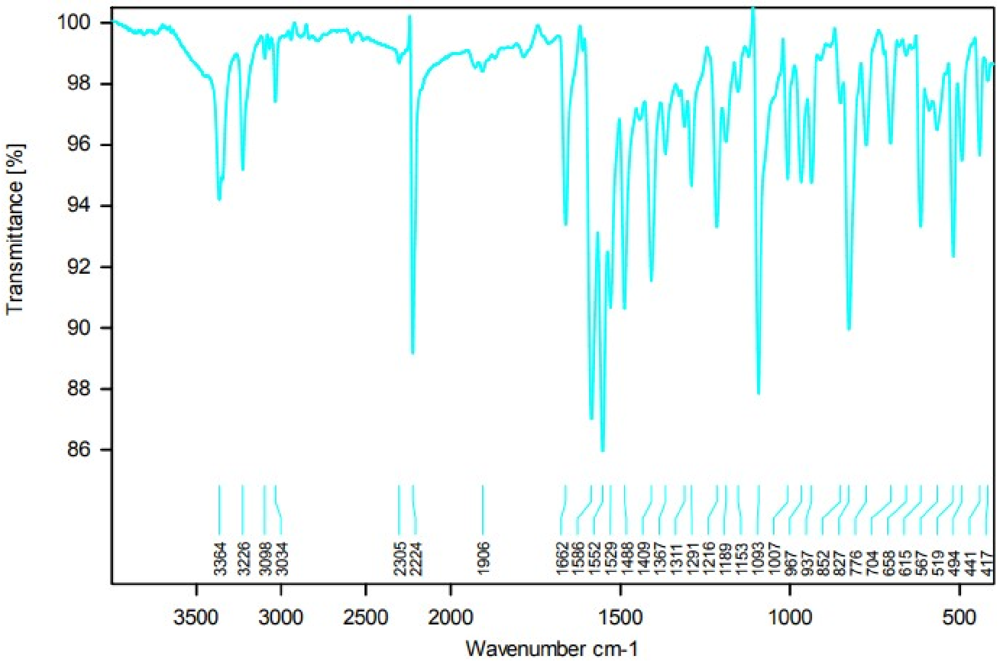

Figure S12. FT-IR spectra of 2-amino-4-(4-chlorophenyl)-6-phenylnicotinonitrile

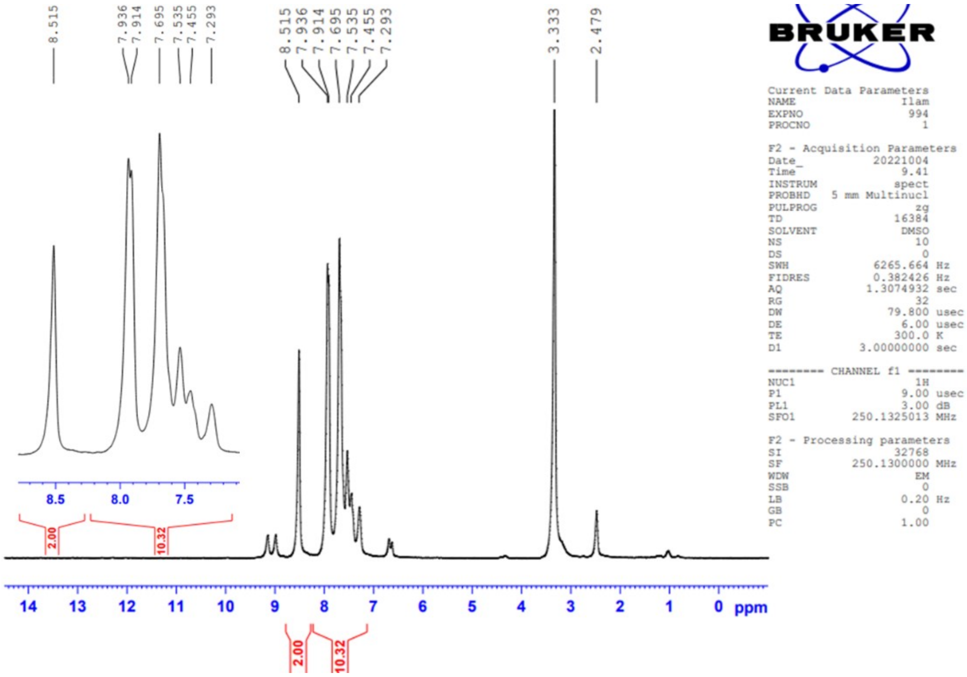

Figure S13. ^{1}HNMR spectra of 2-amino-4-(4-chlorophenyl)-6-phenylnicotinonitrile

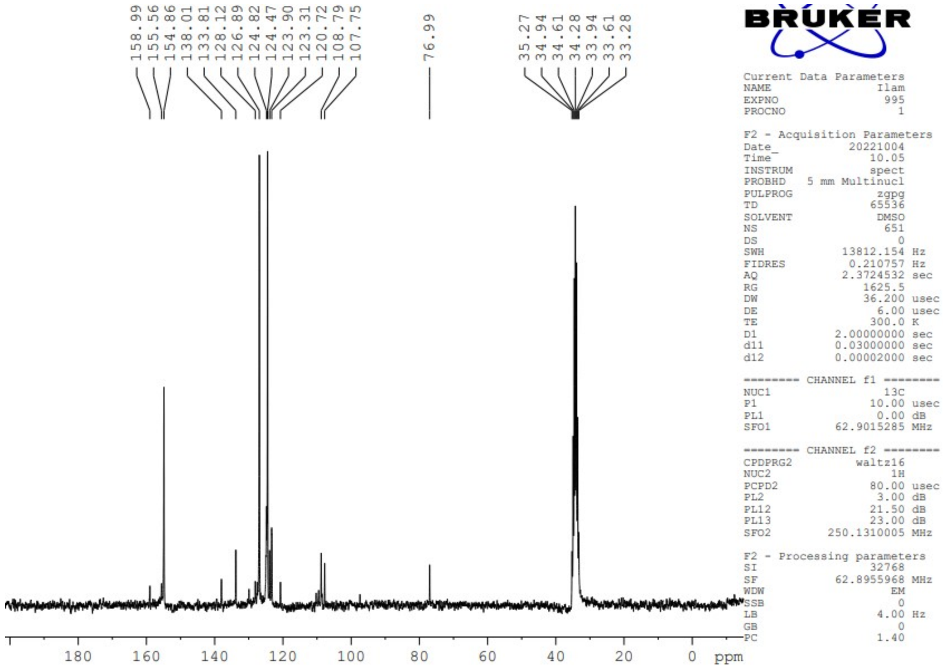

Figure S14. ^{13}CNMR spectra of 2-amino-4-(4-chlorophenyl)-6-phenylnicotinonitrile

5: 2-amino-4,6-diphenylnicotinonitrile (Table 2, 6h, Figure S15-Figure S17): Melting point: 183-185 °C. FT-IR (KBr) ν (cm^{-1}): 3363, 3225, 3056, 2217, 1662, 1552, 967, 752 and 692 cm^{-1}. ^{1}H NMR (250 MHz, DMSO- d_6): δ (ppm)= 9.15-9, 7.43-7.64 (m, 8H), 7.28 (s, 1H), 6.64-6.70 (2H) ^{13}C NMR (63 MHz, DMSO-d_6): δ (ppm)= 159.06, 155.80, 139.52, 129.16, 127.60, 125.48, 124.99, 123.93, 123.32, 122.98, 120.04, 110.28, 109.60, 96.98, 46.37.

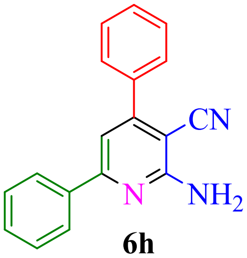

6h

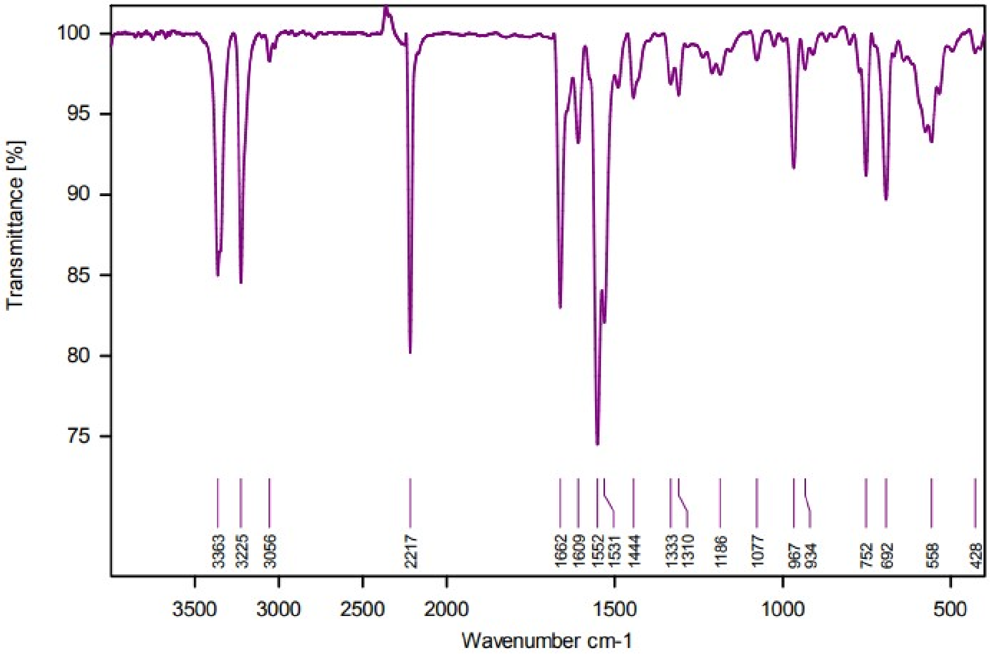

Figure S15. FT-IR spectra of 2-amino-4,6-diphenylnicotinonitrile

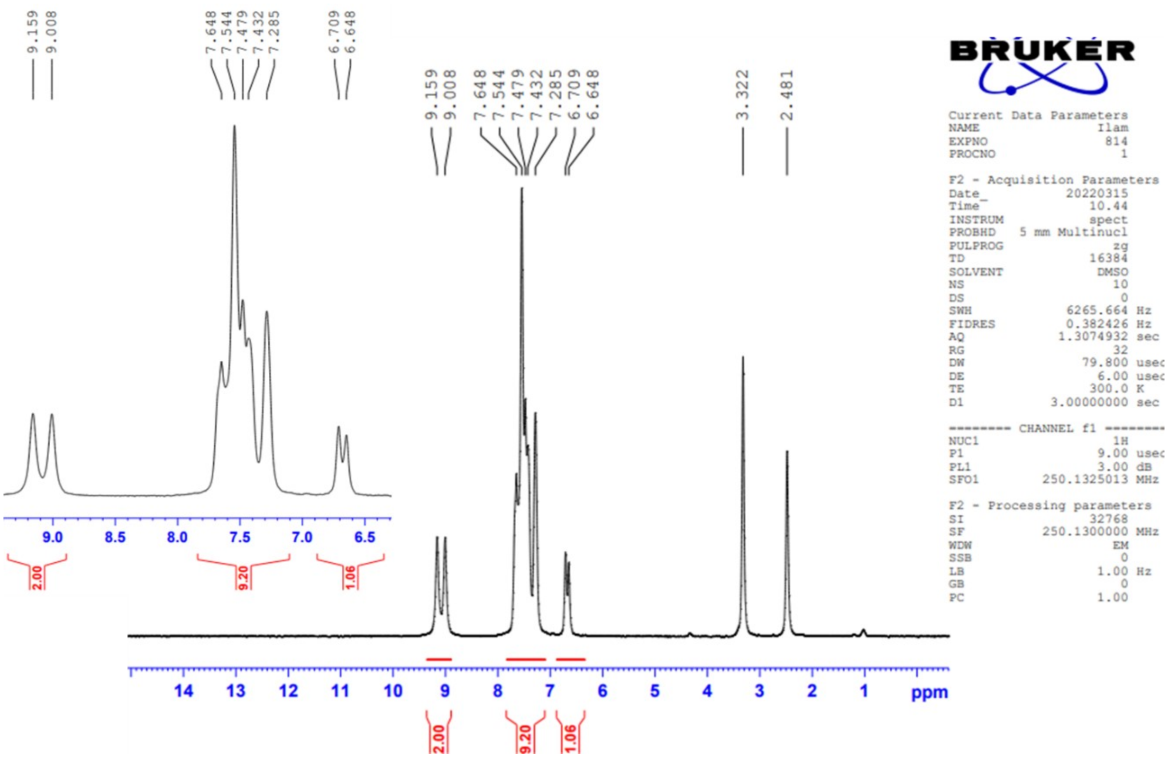

Figure S16. ^{1}HNMR spectra of 2-amino-4,6-diphenylnicotinonitrile

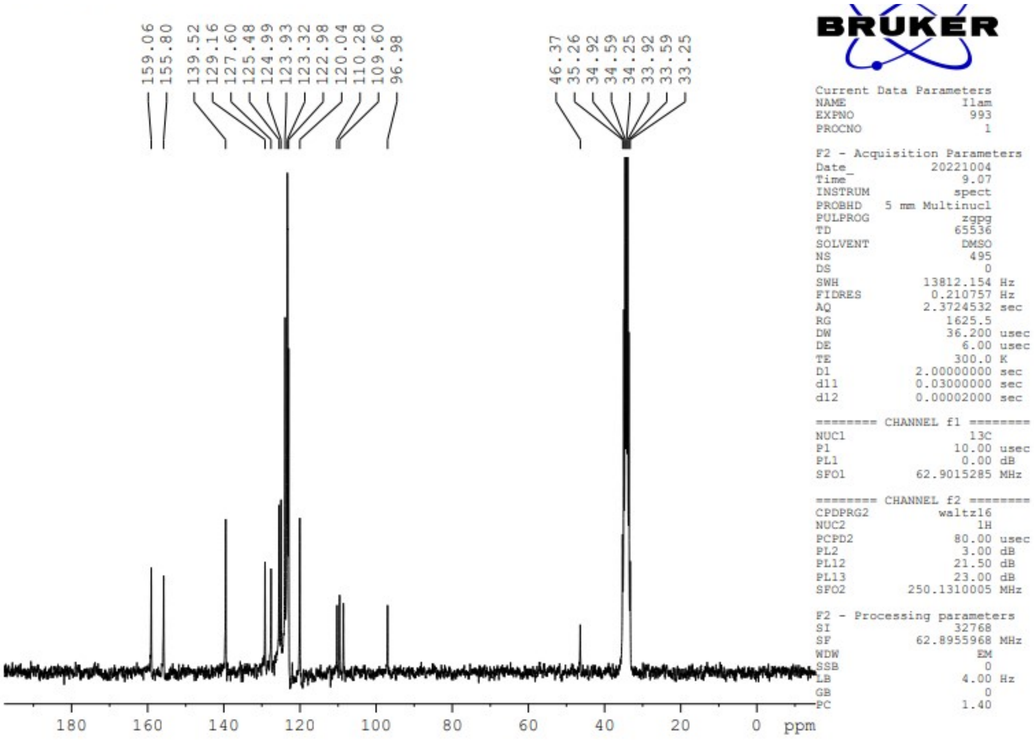

Figure S17. ^{13}CNMR spectra of 2-amino-4,6-diphenylnicotinonitrile

6: 2-amino-6-(4-methoxyphenyl)-4-phenylnicotinonitrile (Table 4, 6j, Figure S18-Figure S20): Melting point: 168-170 °C. FT-IR (KBr) ν (cm^{-1}): 3487, 3368, 2203, 1617, 1578, 1237, 1171, 1026, 952, 829, 765, 699 and 516 cm^{-1}. ^{1}H NMR (250 MHz, DMSO- d_6): δ (ppm)= 6.92 (s, 2H, NH_2), 6.96-8.10 (12H, aromatic), 3.79 (s, 3H, CH_3). ^{13}C NMR (63 MHz, DMSO-d_6): δ (ppm)= 155.76, 155.58, 153.07, 149.42, 131.91, 124.66, 124.25, 123.60, 123.46, 123.06, 111.09, 108.77, 103.23, 80.47, 50.06.

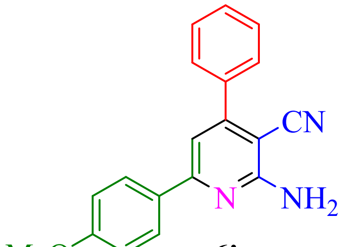

6j

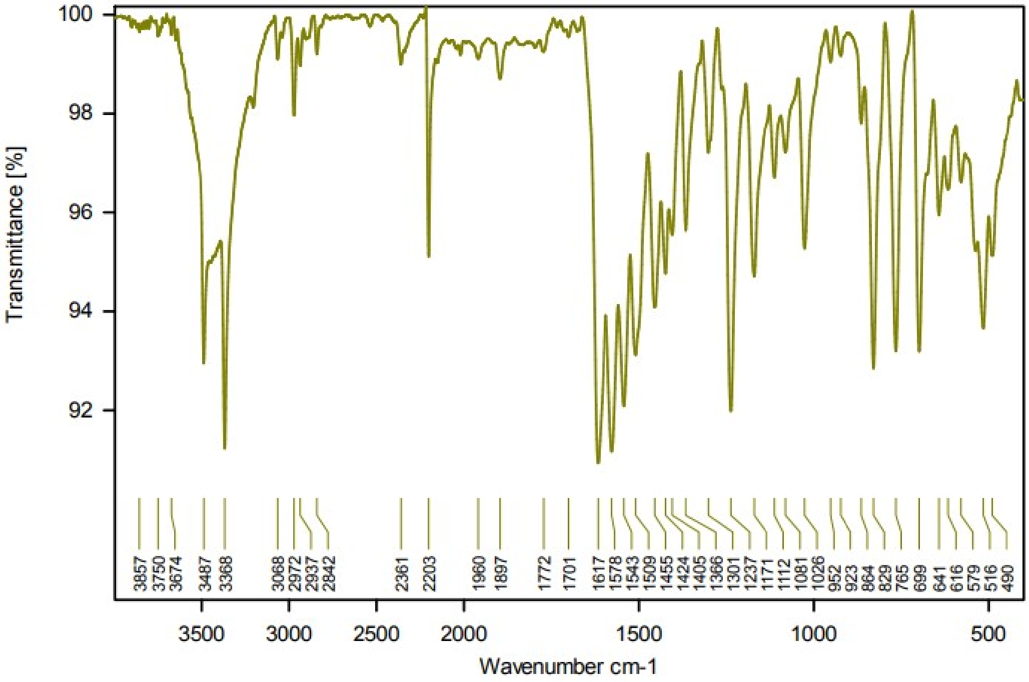

Figure S18. FT-IR specra of 2-amino-6-(4-methoxyphenyl)-4-phenylnicotinonitrile

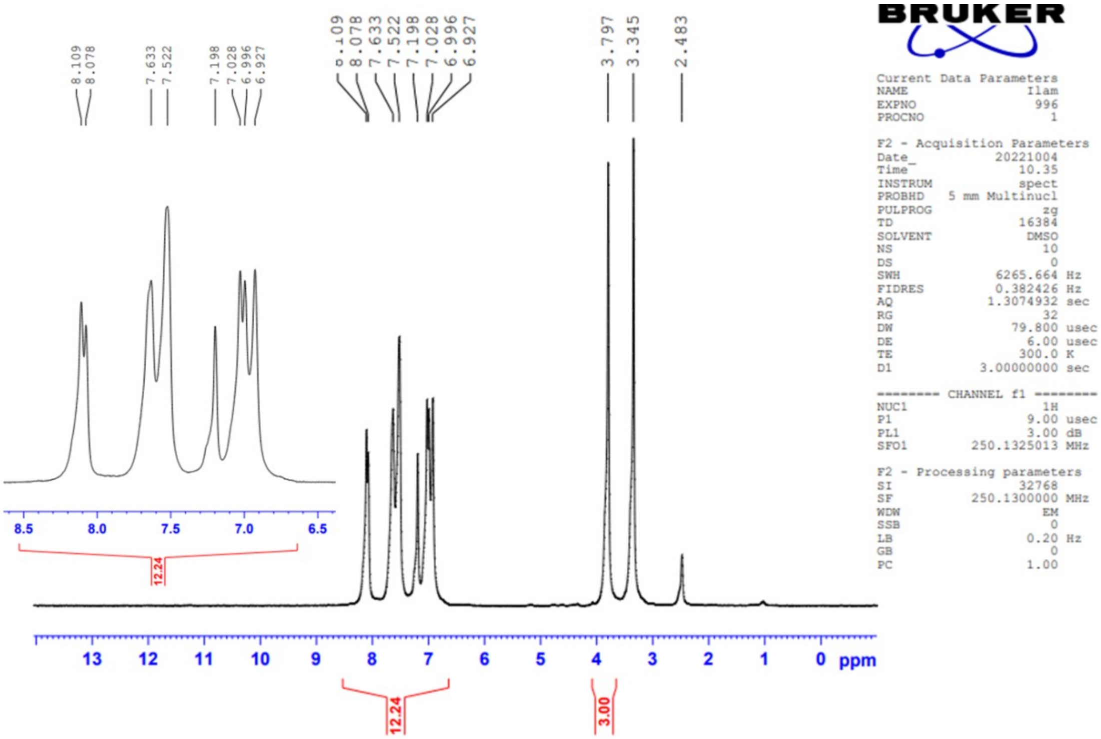

Figure S19. ^{1}H NMR specra of 2-amino-6-(4-methoxyphenyl)-4-phenylnicotinonitrile

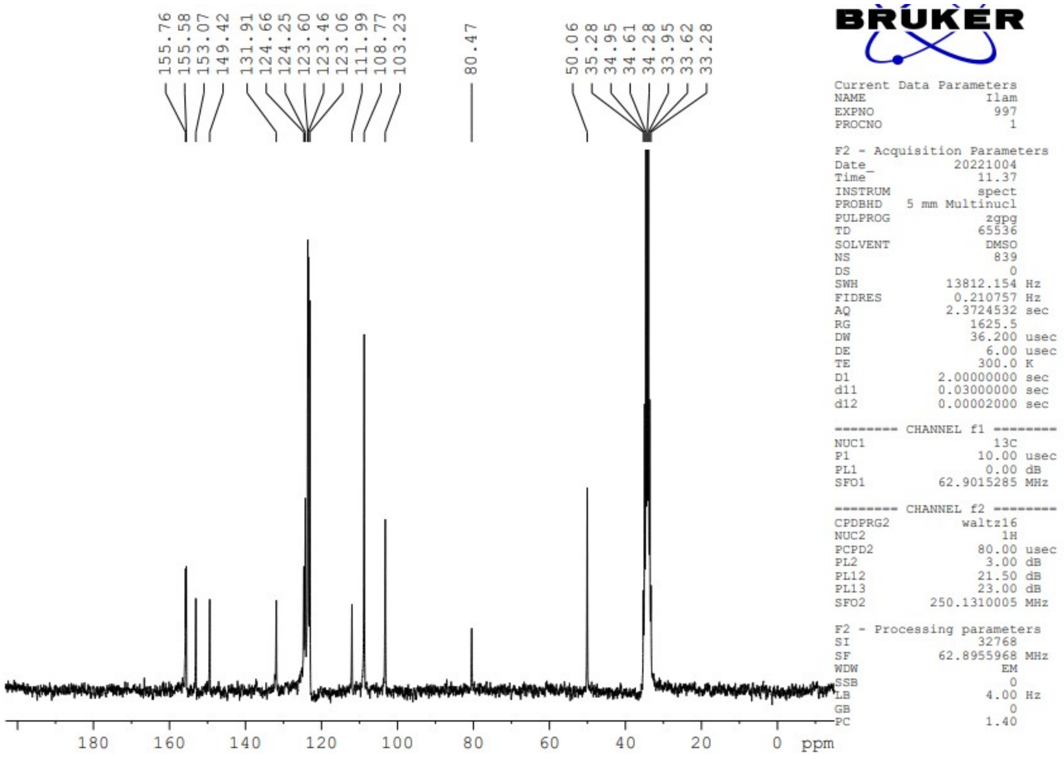

Figure S120. ^{13}CNMR specra of 2-amino-6-(4-methoxyphenyl)-4-phenylnicotinonitrile
